# High-Throughput Time-Lapse Fluorescence Microscopy Screening for Heterogeneously Expressed Genes in Bacillus subtilis

**DOI:** 10.1128/spectrum.02045-21

**Published:** 2022-02-16

**Authors:** Julien Mortier, Stefanie Van Riet, Diana Senovilla Herrero, Kristof Vanoirbeek, Abram Aertsen

**Affiliations:** a Department of Microbial and Molecular Systems, KU Leuven, Leuven, Belgium; Griffith University

**Keywords:** *Bacillus subtilis*, population heterogeneity, gene expression, time-lapse fluorescence microscopy

## Abstract

Elucidating phenotypic heterogeneity in clonal bacterial populations is important for both the fundamental understanding of bacterial behavior and the synthetic engineering of bacteria in biotechnology. In this study, we present and validate a high-throughput and high-resolution time-lapse fluorescence microscopy-based strategy to easily and systematically screen for heterogeneously expressed genes in the Bacillus subtilis model bacterium. This screen allows detection of expression patterns at high spatial and temporal resolution, which often escape detection by other approaches, and can readily be extrapolated to other bacteria. A proof-of-concept screening in B. subtilis revealed both recognized and yet unrecognized heterogeneously expressed genes, thereby validating the approach.

**IMPORTANCE** Differential gene expression among isogenic siblings often leads to phenotypic heterogeneity and the emergence of complex social behavior and functional capacities within clonal bacterial populations. Despite the importance of such features for both the fundamental understanding and synthetic engineering of bacterial behavior, approaches to systematically map such population heterogeneity are scarce. In this context, we have elaborated a new time-lapse fluorescence microscopy-based strategy to easily and systematically screen for such heterogeneously expressed genes in bacteria with high resolution and throughput. A proof-of-concept screening in the Bacillus subtilis model bacterium revealed both recognized and yet unrecognized heterogeneously expressed genes, thereby validating our approach.

## INTRODUCTION

While bacteria grow into a clonal population of isogenic siblings, it has become clear that such siblings can display a distinct individuality regarding their phenotypic behavior, often even irrespective of environmental fluctuations or local gradients (1–3). Such phenotypic heterogeneity among siblings allows for functional differentiation and intricate social behavior, and broadens the adaptive potential of the clonal population as a whole (4). Indeed, bacterial populations have evolved complex regulatory networks that impose heterogeneous gene expression in order to establish bet-hedging strategies that increase fitness in fluctuating environmental conditions by forcing a subset of individuals to invest in stress resistance instead of growth (1, 3, 5). A striking example of this is the process of sporulation in Bacillus subtilis, in which, upon nutrient limitation, only a subpopulation differentiates into recalcitrant endospores, while another subpopulation switches to alternative metabolites to continue growth (6). In addition, phenotypic heterogeneity has the potential to serve as a division-of-labor strategy (1, 3, 4, 7, 8), in which certain costly tasks that benefit the entire population are carried out only by a subset of cells. An example of this was found in stationary phase B. subtilis cultures, where only a proportion of cells produces and secretes the protease subtilisin E, while the entire population is expected to benefit from the freely diffusible degradation products (7).

Next to a fundamental biological understanding of microorganisms, phenotypic heterogeneity is also becoming increasingly relevant for biotechnology and synthetic biology. Indeed, in order to create populations with highly predictable and productive behavior, endogenous genetic circuits supporting unwanted differential performance among siblings need to be recognized and removed (9). On the other hand, it may be beneficial for some applications to capitalize on bet-hedging and division-of-labor strategies to increase robustness and versatility (10–12). As such, a thorough understanding of phenotypic heterogeneity strategies would support their proper implementation in synthetic biology and could unlock more complex functionalities in engineered populations.

Despite its importance for both biology and synthetic biology, current examples of bacterial phenotypic heterogeneity are still limited and typically stem from serendipitous discoveries rather than from systematic screens. The few studies that have engaged in systematic screening for noisy promoter behavior in Escherichia coli, Salmonella typhimurium, and Saccharomyces cerevisiae relied on flow cytometry analysis of fluorescent promoter-probe libraries, and thereby found a positive correlation between the strength and noisiness of promoters (13–15) as well as a tendency for functionally important genes to display low noise (14). Also, single-cell transcriptomic approaches will soon mature and find their way toward revealing population heterogeneity in bacteria (16).

In contrast to flow cytometry and single-cell transcriptomics, however, time-lapse fluorescence microscopy (TLFM) could offer a higher resolution when screening for heterogeneously expressed promoters. Indeed, TLFM monitoring of growing microcolonies of fluorescent promoter-probe clones could reveal more subtle and intricate patterns and timing of heterogeneous promoter activity, while concurrently monitoring important cellular characteristics such as cell morphology, pole age, lineage history, viability, etc. Because of the need for such more detailed approaches for unraveling genetic networks engaged in causing phenotypic heterogeneity, we here present and validate a high-throughput TLFM-based strategy for elucidating deviant promoter expression patterns in the B. subtilis model bacterium.

## RESULTS AND DISCUSSION

### Construction of fluorescent promoter-probe transposon.

**FIG 1:**
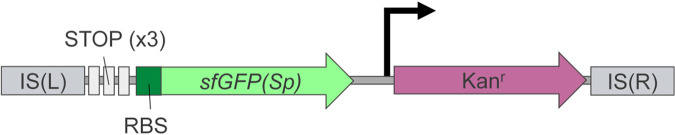
Conceptual scheme of the TnJM1 transposon. The TnJM1 transposon is flanked by two *mariner* insertion sequences, IS(L) and IS(R), which are recognized for random and efficient cut-and-paste transposition into dinucleotide TA recognition sites in the chromosome by the Himar1 transposase (17). The transposon contains a gene encoding a bright low-noise superfolder GFP-variant, *sfgfp(Sp)* (20) with a strong ribosome binding (RBS), for efficient translation when inserted downstream of random chromosomal promoters. Stop codons (STOP) in all three reading frames are located upstream of *sfgfp(Sp)* to avoid the creation of aberrant fusion proteins. A kanamycin antibiotic resistance cassette (Kan^r^) is present as a marker for transposon insertion (the bent arrow represents the promoter for the Kan^r^ cassette). Random TnJM1 transposition in chromosomal open reading frames (ORFs) allows the creation of transcriptional fluorescent fusions that can be monitored with time-lapse fluorescence microscopy. The different elements in the scheme are not drawn to scale.

To systematically screen for heterogeneously expressed promoters in Bacillus subtilis, we designed a novel *mariner*-based transposon, TnJM1 (Fig. 1), that can generate random transcriptional fluorescent fusions upon hopping in the B. subtilis chromosome. *Mariner* transposons are ideally suited for random transposon mutagenesis because chromosomal insertion is highly random (i.e., the very common dinucleotide insertion site TA prevents bias toward chromosomal hot spots) and very efficient (17–19). TnJM1 was constructed by inserting the promoter-less *sfgfp(Sp)* fluorescent reporter gene into a previously described *mariner* transposon that harbors a kanamycin resistance marker (17) (Fig. 1). The *sfgfp(Sp)* gene is optimized for low-GC content Gram-positive bacteria, and codes for a bright superfolder GFP variant with low inherent phenotypic noise strength (20). In TnJM1, *sfgfp(Sp)* was fitted upstream with (i) a strong ribosome binding site for optimal expression (R0 (21)), and (ii) stop codons in all three reading frames to prevent read-through of possible upstream open reading frames. Subsequently, pKB176-TnJM1 was constructed by cloning TnJM1 into an empty pKB176 delivery vector (17, 18), which contains an E. coli origin of replication, the hyperactive C9 allele of the Himar1 transposase, a temperature-sensitive B. subtilis origin of replication, a B. subtilis erythromycin resistance cassette, and an E. coli ampicillin resistance cassette, and was transformed into B. subtilis strain PS832 (further referred to as PS832).

### Construction of fluorescent B. subtilis promoter-probe library.

A library of random promoter fusions with sfGFP(Sp) was subsequently constructed by growing multiple independent PS832 pKB176-TnJM1 cultures for 10 h to allow Himar1-mediated cut-and-paste transposition of TnJM1 into the chromosome. These multiple cultures were pooled to reduce the impact of clonal amplification within a population, and then plated on kanamycin selective LB agar plates and incubated at 42°C. The latter temperature is nonpermissive for maintenance of the delivery vector due to the temperature-sensitive origin of replication and will therefore select for clones in which TnJM1 transposed into the chromosome. A minority of kanamycin resistant clones (5.67%; SE = 0.67%) was found to remain erythromycin resistant, suggesting the entire delivery vector stably inserted itself into their chromosome. However, these clones were systematically excluded from the screening process by replica plating library clones on erythromycin selective plates and disregarding the erythromycin resistant ones.

### High-throughput screening of the B. subtilis promoter-probe library.

**FIG 2:**
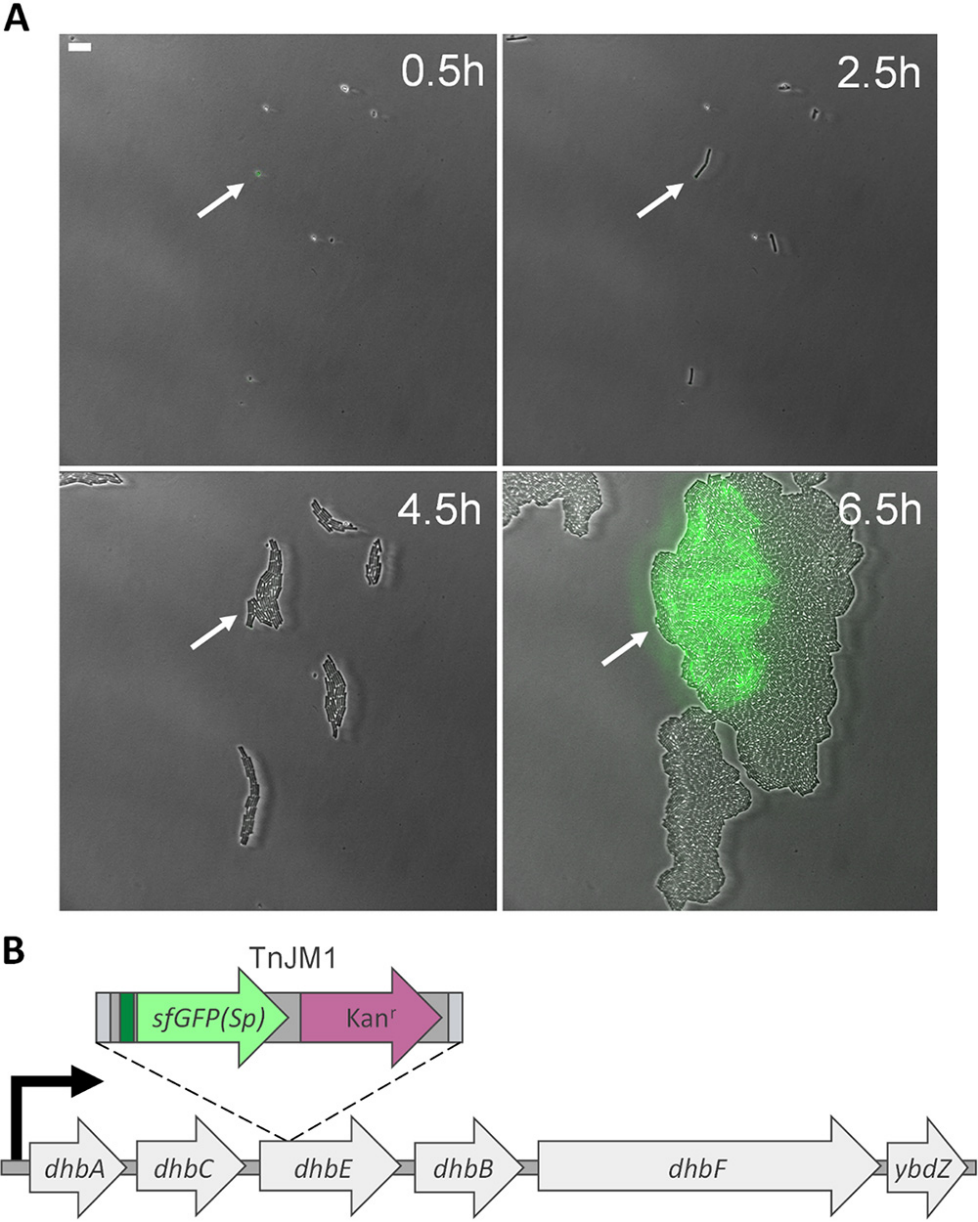
Example of single cell TLFM-screening of library clones. (A) Representative superimposed phase contrast and epifluorescence (reporting sfGFP(Sp) expression from a random chromosomal promoter) images of a pool of 32 library clones growing on a germination-inducing agarose pad at several time points during TLFM. One of the microcolonies in this screen (indicated by the white arrows) developed from a single endospore into a clonal population heterogeneously expressing sfGFP(Sp). Because of its interesting phenotype, this clone was traced back to its original location on the 96-well plate and determined to contain TnJM1 in its *dhbE* gene. Scale bar corresponds to 10 μm. (B) Schematic representation of the TnJM1 insertion in the fluorescent clone shown in panel A. The TnJM1 transposon (containing the sfGFP(Sp) open reading frame [green arrow] and its ribosome binding site [green box], kanamycin antibiotic resistance cassette [Kan^r^; purple arrow] and insertion sequences [gray boxes]; see Fig. 1) was located in the *dhbE* gene within the *dhbACEBF-ybdZ* operon, yielding a fluorescent transcriptional fusion. The bent arrow (black) represents the operon’s promoter. The different elements in the scheme are not drawn to scale.

Individual clones of the library were then grown in 96-well plates and subsequently passaged to fresh wells containing starvation medium to produce spore crops. Endospores were pooled per 32 clones (i.e., one third of a 96-well plate), and pools were heat activated to induce germination and inactivate any remaining vegetative cells. Subsequently, up to 18 separate pools per screen (i.e., encompassing 576 clones) were placed on an equal number of germination-inducing agarose pads (Fig. 2A), and the sfGFP(Sp) expression of single cells in emerging microcolonies was then monitored for 20–24 h using time-lapse fluorescence microscopy (TLFM; Fig. 2A and B). In fact, this allowed close observation of expression patterns over different consecutive life stages of the individual cells/spores, including germination of a single spore, exponential growth into a clonal microcolony, and growth arrest due to starvation (please note that the subsequent sporulation step was not systematically captured; see below). In this fashion, a total of ca. 7,700 individual transposon insertion clones was screened. Since these transposon insertions have stochastically sampled the chromosome, we estimate that these clones transcriptionally report on roughly 55% (± 0.62%; based on Monte Carlo sampling) of the ca. 4,350 annotated B. subtilis genes (22, 23) and their corresponding promoters. Note, however, that not all of these genes might become expressed under the cultivation conditions used.

### Outcome of the screen.

**FIG 3:**
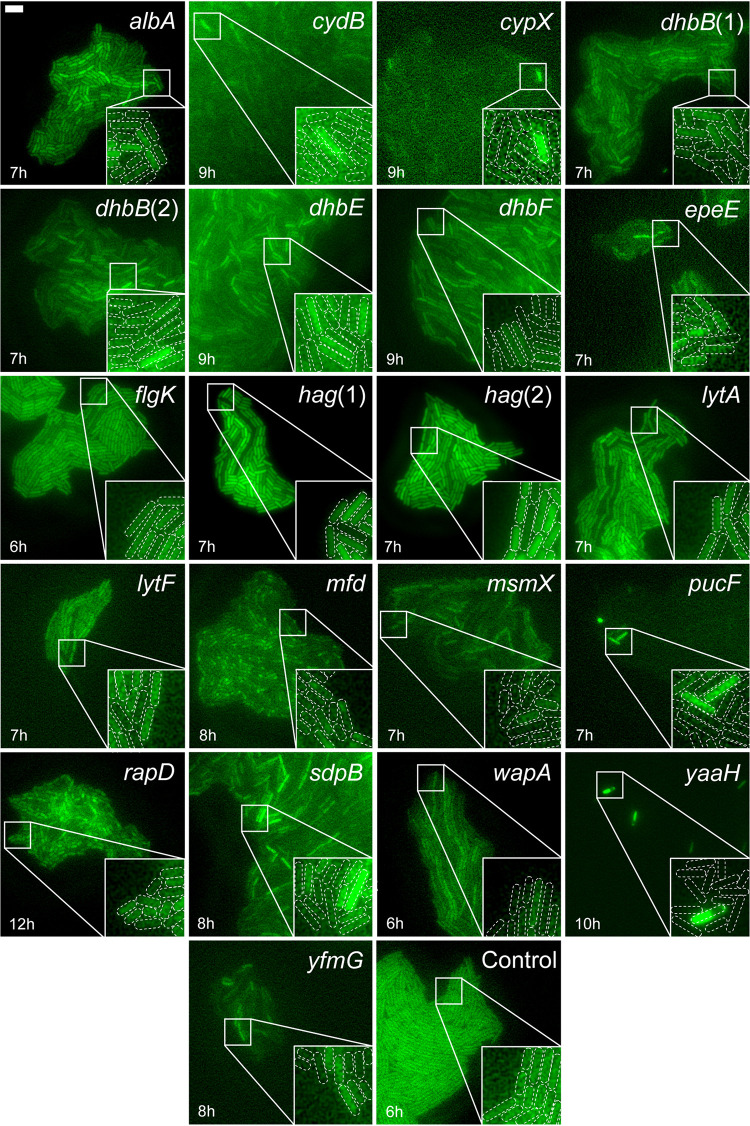
Heterogeneous promoter expression of reconstructed mutants. Representative epifluorescence (reporting sfGFP(Sp) expressed from the TnJM1 transposon inserted into the indicated genes) images of representative microcolonies of reconstructed mutants grown on MOPS agarose pads. As a control, an isolated TnJM1 insertion mutant was added in which sfGFP(Sp) fluorescence is expressed homogeneously among siblings. Images were selected at different time points after being placed on the pads (indicated on figure) to best represent the individual expression patterns. Due to differences in fluorescence expression among the isolated clones, image brightness was adjusted for each mutant separately, and fluorescence intensity can therefore not be compared between mutants. An indicated part of each image is magnified 3 times (inset lower right corner), and individual cells are delineated with white dashed outlines. Scale bar corresponds to 5 μm.

Microcolonies displaying heterogeneous sfGFP(Sp) expression among their isogenic siblings were traced back to their original wells in the master 96-well plate, ultimately yielding 21 independently isolated mutants with heterogeneously expressed *sfgfp(Sp)* promoter fusions (Fig. 3; Movies 1–21 in the supplemental material). After SPP1-mediated transduction to wild-type PS832, the causal individual TnJM1 insertions could be isolated and determined. This revealed heterogeneous expression of 19 different genes corresponding to 17 separate operons (Table 1).

**TABLE 1:** Overview of genes identified as TnJM1 transposon insertion sites in isolated clones with heterogeneous sfGFP(Sp) expression, and (i) their corresponding gene products, (ii) the operon the gene is part of, and (iii) the cellular regulon and function it is involved in (based on de SubtiWiki database [40])

Gene	Gene product	Operon	Known regulons	Function
*albA* ^*a*^	Antilisterial bacteriocin subtilosin biosynthesis protein	*sboA-sboX-albA-albB-albC-albD-albE-albF-albG*	AbrB, σ^A^, Rok, ResD	Subtilosin A biosynthesis
*cydB* ^*a*^	cytochrome bd ubiquinol oxidase (subunit II)	*cydA-cydB-cydC-cydD*	CcpA, Rex, ResD, σ^F^	Respiration
*cypX* ^*a*^	Pulcherriminic acid synthase/cytochrome P450	*yvmC-cypX*	AbrB, PchR	Iron metabolism
*dhbB* ^*a*^ ^,^ ^*b*^	Isochorismatase	*dhbA-dhbC-dhbE-dhbB-dhbF-ybdZ*	σ^A^, σ^I^, Fur, AbrB, Kre, σ^I^	Siderophore bacillibactin biosynthesis
*dhbE* ^*a*^	2,3-dihydroxybenzoate-AMP ligase	*dhbA-dhbC-dhbE-dhbB-dhbF-ybdZ*	σ^A^, σ^I^, Fur, AbrB, Kre, σ^I^	Siderophore bacillibactin biosynthesis
*dhbF* ^*a*^	Dimodular nonribosomal peptide synthase	*dhbA-dhbC-dhbE-dhbB-dhbF-ybdZ*	σ^A^, σ^I^, Fur, AbrB, Kre, σ^I^	Siderophore bacillibactin biosynthesis
*epeE* ^*a*^	Radical SAM epimerase	*epeX-epeE-epeP-epeA-epeB*	AbrB, σ^A^, Rok	Cell envelope stress
*flgK*	Flagellar hook-associated protein 1	*comFA-comFB-comFC-yvyF-flgM-yvyG-flgK-flgL-yviE-fliW-csrA*	σ^A^, σ^D^, DegU, ScoC, ComK	Flagellum biosynthesis
*hag* ^*b*^	Flagellin	*hag*	σ^D^, CodY, ScoC, CsrA	Flagellum biosynthesis
*lytA*	Autolysin-associated protein	*lytA-lytB-lytC*	σ^A^, σ^D^, SlrR, SinR, YvrHb	Peptidoglycan remodeling
*lytF*	Peptidoglycan endopeptidase	*lytF*	σ^D^, SlrR, SinR	Peptidoglycan remodeling
*mfd* ^*a*^	Transcription-repair-coupling factor	*fin-mfd-spoVT*	σ^B^, σ^F^, σ^G^	DNA repair
*msmX* ^*a*^	ATP-binding subunit of ABC transporters	*yxkF-msmX*	CcpA	Carbohydrate transport
*pucF* ^*a*^	Allantoate amidohydrolase	*pucF-pucG*	PucR, σ^A^	Purine utilization
*rapD* ^*a*^	Response regulator aspartate phosphatase	*rapD*	RghR, σ^A^, σ^M^, σ^X^	Controls ComA-dependent gene expression
*sdpB*	Sporulation-delaying protein B	*sdpA-sdpB-sdpC*	Spo0A, Rok, AbrB	SdpC toxin maturation
*wapA* ^*a*^	tRNA nuclease	*wapA-wapI*	DegU, YvrHb, WalR, σ^A^	Contact-dependent growth inhibition (CDI)
*yaaH*	Spore peptidoglycan N-acetylglucosaminidase	*yaaH*	SpoIIID, σ^E^, σ^B^	General stress protein, inner coat protein, germination
*yfmG* ^*a*^	Uncharacterized protein	*yfmH-yfmG*	AbrB, YfmH, σ^A^	Unknown

a To our knowledge, the heterogeneous expression of these genes and/or their corresponding operons have not been previously described in literature.

b A transposon insertion was found in different insertion sites in this gene for two independently isolated clones.

A number of these heterogeneously expressed operons were already documented in (or easily extrapolated from) previous literature, and relate to the intrinsically bistable σ^D^ regulon (*hag*, *comFA-comFB-comFC-yvyF-flgM-yvyG-flgK-flgL-yviE-fliW-csrA*, *lytABC*, *lytF* (24–26)) or genes involved in the process of sporulation (*yaaH*, *sdpABC*, *mfd* [27–32]).

The *hag* and *flgK* genes have been shown to be bistably expressed under the control of the alternative sigma factor σ^D^, and encode the structural protein for the flagellar filament and the protein for the hook-filament junction, respectively (25). By bistable expression, σ^D^ is known to mediate the formation of two distinct subpopulations in midexponential phase, namely, motile (σ^D^ ON) cells and chaining sessile (σ^D^ OFF) cells (24, 25). This bistability is likely achieved through a variety of simultaneously acting mechanisms, including a σ^D^ positive feedback loop (24), a SinR/SlrR-mediated double negative feedback loop (33), and a differential reduction in growth rate causing a concentration effect of σ^D^ (34). The heterogeneity in flagellar gene expression is quite common among bacteria, and serves to exploit their present location or to explore new environmental niches (25, 34–36). Two other genes found during screening, i.e., *lytA* (part of the *lytABC* operon) and *lytF*, are both involved in peptidoglycan-remodeling-autolysin activity, and are also known to be under σ^D^-mediated bistable expression, enabling cell separation and motility in σ^D^ ON subpopulations (26).

The commitment to sporulation is intrinsically noisy because of the multicomponent phosphorelay that activates the master regulator of sporulation Spo0A (37). Moreover, this noisiness serves as a bet-hedging strategy. Upon nutrient limitation, part of the population will initiate sporulation when Spo0A∼P reaches a high threshold level (38), while the subpopulation in which Spo0A is not active will not sporulate. The decision to sporulate can be postponed by cannibalism, which enables the Spo0A active subpopulation to grow by use of alternative metabolites released by inducing lysis of the nonsporulating subpopulation (6, 30). Accumulation of Spo0A∼P activates a signaling cascade of sigma factors, of which some are specific to the forespore (σ^F^, σ^G^) and some are specific to the mother cell compartment (σ^E^, σ^K^) (39). As such, we found localized gene expression specific to the forespore (*mfd* [σ^F^/σ^G^ dependent (32)]; Fig. 3 and Movies 14A and B) and mother cell (*yaaH* [σ^E^ dependent (28)]; Fig. 3 and Movies 20A and B). Interestingly, while the *cydABCD* operon has been described to be in part controlled by forespore-specific σ^F^ (40, 41), the observed expression pattern for this operon does not reveal any forespore-specific expression and localization (Fig. 3 and Movies 2A and B). Since we did not systematically allow the monitored clones to enter the sporulation phase, only a fraction of the many heterogeneously expressed sporulation-related genes were picked up.

Interestingly, and more unexpectedly, several AbrB-dependent promoters (*sboAX-albABCDEFG*, *yvmC-cypX*, *dhbACEBFZ*, *yfmHG*, *epeXEPAB*) were picked up as being heterogeneously expressed as well. AbrB is a global gene regulator involved in transitioning the cell from exponential to stationary phase, and cell-to-cell variations in AbrB protein levels have been documented (42) that could perhaps explain the heterogeneous expression of the AbrB-dependent promoters. Interestingly, there seem to be stark differences in timing and heterogeneity of the expression patterns of these promoters (Fig. 3; Movies 1A, 1B, 3A, 3B, 4A, 4B, 5A, 5B, 6A, 6B, 7A, 7B, 8A, 8B, 21A, and 21B), possibly due to modulation by other regulatory factors. While the AbrB regulator is known to be under negative Spo0A control (43, 44), the timing of heterogeneous gene expression by some AbrB-dependent promoters found in this screen (e.g., *sboAX-albABCDEFG*; Fig. 3; Movies 1A and B) already occurs in the exponential phase and does therefore not seem to coincide with the expected Spo0A-related (i.e., stationary phase) heterogeneity. As such, while Spo0A noisiness may be a modulating factor, it seems unlikely that it forms the sole causative basis for these heterogeneous expression patterns.

Also, several other genes were previously not known (or anticipated) to be heterogeneously expressed. One of these, *rapD*, has a clear regulatory role in the inhibition of the response regulator ComA, which is involved in the development of natural competence and quorum sensing (40, 45). While the regulation of the bistable master regulator of competence (i.e., ComK) is complex and includes an auto-stimulatory feedback loop (46), *rapD* may provide an additional form of heterogeneity into this regulatory system. Also, several metabolically oriented operons (*cydABCD*, *pucFG*, *yxkF-msmX*) seem to be heterogeneously expressed. While CydB and PucF are involved in aerobic respiration and nitrogen metabolism, respectively (47, 48), MsmX acts as the nucleotide binding domain for multiple ABC transporter complexes that mediate the import of several carbohydrates (including maltodextrin, melibiose, raffinose, and stachyose [49, 50]) and the mobilization of pectin (51). Finally, *wapA* is involved in contact-dependent growth inhibition (52).

### Conclusions.

In this report, we forwarded and validated a novel high-throughput approach to systematically screen for heterogeneously expressed genes/promoters in bacteria at high spatial and temporal resolution, since such important features within clonal populations currently typically evade our detection. The approach is based on a randomly transposable fluorescent promoter probe and high-throughput TLFM-monitoring, and is therefore amenable to a wide range of bacterial hosts. Our library fluorescently reported on ca. 55% of the genes present in B. subtilis and found both recognized and still unrecognized heterogeneously expressed promoters, validating the potential of our approach. Imposing different cultivation conditions during the screen will of course alter the subset of genes being expressed and hence the heterogeneous promoters that can be detected. A potential caveat of our approach, however, is that in some rare cases, insertion of the reporter transposon could alter the normal expression profile of the probed gene.

Since B. subtilis constitutes an important chassis in bioprocessing (53, 54), identifying (and subsequently eliminating) possible causes of behavioral heterogeneity and inconsistencies within axenic populations becomes increasingly important. On the other hand, novel synthetic biology tools aimed at deliberately imposing population heterogeneity in a standardized fashion will soon depend on identifying (and characterizing) noisy and bistably expressed promoters.

## MATERIALS AND METHODS

### Bacterial strains and growth conditions.

Bacterial strains and plasmids are listed in Tables S1 and S2, respectively. For strain construction, Lysogeny Broth (LB) according to Lennox (10g/L tryptone [Lab M, Lancashire, United Kingdom], 5g/L yeast extract [Oxoid, Hampshire, United Kingdom], 5g/l NaCl) was used. For growth for transposon library construction, time-lapse microscopy (TLM) medium and 15% chemically defined medium (CDM) were used as previously described (6, 55). MOPS medium (adapted from Kort et al. [56]) was used for agarose pads in TLFM and contains 1.32 mM K_2_HPO_4_, 0.4 mM MgCl_2_, 0.276 mM K_2_SO_4_, 0.01 mM FeSO_4_, 0.14 mM CaCl_2_, 80 mM 3-[N-morpholino]propanesulfonic acid (MOPS), 4 mM Tricine, 10 mM NH_4_Cl, 3 nM (NH_4_)_6_Mo_7_O_24_, 0.4 μM H_3_BO_3_, 30 nM CoCl_2_, 10 nM CuSO_4_, 10 nM ZnSO_4_, 0.1 mM MnCl_2_, 27.8 mM glucose (Acros Organics, Geel, Belgium), 0.02% Casamino Acids (LabM) and supplemented with 30 mM l-valine (Fisher Scientific, Pittsburgh, PA, USA) where indicated. This medium was selected for its high germination efficiency and low background fluorescence. SP medium (phosphate citrate buffer [14g/l K_2_HPO_4_, 6g/l KH_2_PO_4_, 1g/l sodium citrate with 2% glucose, 0.1% Casamino acids, 0.05 mg/mL L-tryptophan, 0.011 mg/mL CAF (ferric ammonium citrate), 0.2% potassium aspartate and 3 mM MgSO_4_]) was used to make naturally competent B. subtilis cells.

When appropriate, the following antibiotics were added to the medium at the indicated final concentrations: 100 μg/mL ampicillin (Fisher Scientific, Pittsburgh, PA, USA; E. coli), 5 μg/mL kanamycin (Applichem, Darmstadt, Germany; B. subtilis), 1 μg/mL erythromycin (Acros Organics; B. subtilis).

### B. subtilis transformation.

Cultures of B. subtilis PS832 were made naturally competent by picking a colony from a fresh stock plate and growing it overnight at 30°C in 3 mL of SP medium. The following morning, the culture was diluted 1/50 by adding 200 μl to 10 mL of fresh SP medium. The culture was incubated at 30°C until an OD_600_ of 0.6–0.8 was reached, indicating that cells were ready to be transformed. Plasmid transformation was achieved by adding 5 μL of plasmid (concentration between 50 and 150 ng/μL) to 500 μL of competent cells. Cells were resuscitated at 30°C for 0.5–1 h in culture tubes before plating on selective media. Plates were incubated overnight at 30°C.

### TnJM1 transposon construction.

To construct the TnJM1 transposon, an amplicon of *sfgfp(Sp)* (20) was prepared on pDR111-sfgfp(Sp) with primers P1 and P2 (Table S3), introducing restriction sites for SalI and BamHI. Using these restriction sites, *sfgfp(Sp)* was inserted in the *mariner* transposon in pKB157 through restriction/ligation, creating the pKB157-TnJM1 plasmid, which was transformed into electrocompetent Escherichia coli DH5α cells. An amplicon containing TnJM1 was amplified from this plasmid using primer pair P9/P10 (Table S3), digested with PstI and HindIII and ligated into the delivery vector pKB176 to generate pKB176-TnJM1. This plasmid was initially transformed into E. coli DH5α and subsequently transformed into B. subtilis PS832.

All constructed plasmids were initially confirmed by PCR with primer pairs attaching outside of the region of insertion (P17-54; Table S3). Correct insertion of digested PCR products was further verified by sequencing (Macrogen, Amsterdam, the Netherlands).

### Library construction.

To construct a library of random promoter fusions with *sfgfp(Sp)*, through transposon mutagenesis of TnJM1, 7 independent cultures of B. subtilis PS832 pKB176-TnJM1 were grown from glycerol stocks for 12 h at 30°C in 4 mL LB medium supplemented with erythromycin (selecting for maintenance of the pKB176-TnJM1 delivery vector). Subsequently, these cultures were plated on LB agar supplemented with kanamycin (selecting for presence of TnJM1) and incubated overnight at 42°C (counterselecting against the presence of the pKB176-TnJM1 delivery vector). The frequency of false positive insertions of the delivery vector into the chromosome was tested for three independently made libraries by streaking 100 library clones on both LB agar containing erythromycin and LB agar containing kanamycin, and assessing growth after overnight incubation at 30°C.

To exclude false positive clones that have not lost the pKB176-TnJM1 delivery vector, replica plating (using sterile Whatman filter papers [GE Healthcare, Chicago, IL, USA]) was performed on LB agar supplemented with erythromycin (indicating the presence of the delivery vector). From the LB agar plates containing kanamycin, individual erythromycin-sensitive clones were manually picked into 96-well plates containing 150 μL TLM medium (55) (supplemented with kanamycin) per well, and grown overnight at 37°C in an orbital shaker (225 rpm). By adding glycerol to a final concentration of 20%, these master plates could be stored at −80°C to isolate clones of interest after screening.

### Screening and isolating clones of interest.

Cultures grown in TLM were diluted 1/25 in 96-well plates containing 150 μL of previously described CDM sporulation medium (diluted to 15% before use [6, 55]) per well, and incubated at 37°C in an orbital shaker (225 rpm) for 72h, during which sporulation occurs. Subsequently, spores were pooled per 32 wells and 20 μL of pooled spore suspension was heat activated in a heating block (70°C, 30 min) to induce germination and inactivate vegetative cells.

For TLFM screening, appropriate dilutions of spore suspension pools were transferred to individual agarose pads containing MOPS medium supplemented with l-valine to induce germination. Germination, growth, and sfGFP(Sp) expression on agarose pads was monitored using an automated TLFM set-up for 20–24 h at 37°C. Routinely, during one such 20–24 h TLFM screening event, ca. 18 agar pads (each supporting a pool of 32 clones, amounting to ca. 576 different clones) could be simultaneously monitored. Per agarose pad 5 fields were imaged to monitor a sufficient number of spores.

Fast backtracing of clones of interest to their individual wells on the 96-well master plates was achieved by simultaneously dividing the original plate into smaller subpools per row and column and monitoring these subpools again for the phenotype of interest using TLFM. The row and column subpools containing the correct phenotype reveals the exact coordinates of the clone of interest on the 96-well master plate.

Subsequently, all isolated clones of interest were purified from the master plates by streaking on LB agar (supplemented with kanamycin). To avoid continuing with mutants possibly containing multiple TnJM1 insertions, SPP1 phage lysates of the originally isolated mutants were transduced to a fresh PS832 wild-type strain, and the resulting transducants were again monitored using TLFM for the phenotypes of interest during growth on MOPS agarose pads (supplemented with l-valine) starting from endospores (spore suspensions prepared in TLM and CDM as described above). SPP1 phage transduction was performed as previously described (57).

### Time-lapse fluorescence microscopy (TLFM).

Appropriate dilutions of cultures or spore suspensions were placed on agarose pads (MOPS medium supplemented with 1.5% LSL-LE 8200 agarose [Lonza, Basel, Switzerland] and 30 mM l-valine) on a microscopy slide and covered with a cover glass attached to a 125 μL Gene Frame (Thermo Fisher Scientific, Waltham, MA, USA). The process of making agarose pads has been previously described in more detail by De Jong et al. (55). Automated TLFM monitoring was performed on a widefield Ti-Eclipse inverted microscope (Nikon, Champigny-sur-Marne, France) equipped with a 60× Plan Apo λ oil objective, a TI-CT-E motorized condenser, and a Nikon DS-Qi2 camera. GFP was imaged using a quad-edge dichroic (395/470/550/640 nm) and a FITC single emission filter. A SpectraX LED illuminator (Lumencor, Beaverton, OR, USA) was used as light source, using the 470/24 excitation filter. Temperature was controlled at 37°C with an Okolab cage incubator (Okolab, Ottaviano, Italy). While phase contrast images were taken every 15 min, GFP was imaged every 30 min in order to avoid bleaching. Images were acquired using NIS-Elements software (Nikon), and the resulting pictures were further handled with the open source software ImageJ. During acquisition of fluorescent images, photobleaching was reduced by lowering the intensity of excitation light and prolonging time intervals between exposures.

### Monte Carlo simulations.

An occurrence of 7,700 random transposon insertions in 4,350 B. subtilis genes (considering both possible orientations of the transposon) was simulated 5,000 times to calculate the approximate proportion of genes covered with transposon insertions that yield transcriptional fusions. This also allowed the calculation of the standard deviation of this proportion. Note that this estimate does not take into account that transposon insertions can also occur in noncoding DNA.

### Determination of TnJM1 insertion sites.

To determine the precise genomic location of TnJM1 insertions, the genomic DNA of the clones of interest was pooled per 5 and analyzed by whole genome sequencing. For this, genomic DNA was isolated from overnight LB cultures using the GeneJet Genomic DNA purification kit (Thermo Fisher Scientific), after which 150 bp paired-end libraries were prepared using the Nextera XT DNA Library Preparation Kit (Illumina) or Nextera DNA Flex Library Preparation Kit (Illumina). Sequencing was performed with an Illumina NovaSeq 6000 sequencer (Illumina) or Miniseq sequencer (Illumina), and single reads were analyzed using Geneious to determine TnJM1 insertion loci. All genomic transposition sites were confirmed by PCR using primers upstream and downstream of the suspected region of interest (Table S3) and by subsequent sequencing of this locus (Macrogen). Sequences of the TnJM1 insertions sites for the different isolated mutants are listed in supplemental file 1.

## References

[1] Ackermann M. 2015. A functional perspective on phenotypic heterogeneity in microorganisms. Nat Rev Microbiol 13:497–508. doi:10.1038/nrmicro3491. 26145732

[2] Davis KM, Isberg RR. 2016. Defining heterogeneity within bacterial populations via single cell approaches. Bioessays 38:782–790. doi:10.1002/bies.201500121. 27273675

[3] Mortier J, Tadesse W, Govers SK, Aertsen A. 2019. Stress-induced protein aggregates shape population heterogeneity in bacteria. Curr Genet 65:865–869. doi:10.1007/s00294-019-00947-1. 30820637

[4] West SA, Cooper GA. 2016. Division of labour in microorganisms: an evolutionary perspective. Nat Rev Microbiol 14:716–723. doi:10.1038/nrmicro.2016.111. 27640757

[5] Veening J-W, Smits WK, Kuipers OP. 2008. Bistability, epigenetics, and bet-hedging in bacteria. Annu Rev Microbiol 62:193–210. doi:10.1146/annurev.micro.62.081307.163002. 18537474

[6] Veening J-W, Stewart EJ, Berngruber TW, Taddei F, Kuipers OP, Hamoen LW. 2008. Bet-hedging and epigenetic inheritance in bacterial cell development. Proc Natl Acad Sci USA 105:4393–4398. doi:10.1073/pnas.0700463105. 18326026 PMC2393751

[7] Veening J-W, Igoshin OA, Eijlander RT, Nijland R, Hamoen LW, Kuipers OP. 2008. Transient heterogeneity in extracellular protease production by *Bacillus subtilis*. Mol Syst Biol 4:184. doi:10.1038/msb.2008.18. 18414485 PMC2387230

[8] Arnoldini M, Vizcarra IA, Peña-Miller R, Stocker N, Diard M, Vogel V, Beardmore RE, Hardt W-D, Ackermann M. 2014. Bistable expression of virulence genes in salmonella leads to the formation of an antibiotic-tolerant subpopulation. PLoS Biol 12:e1001928. doi:10.1371/journal.pbio.1001928. 25136970 PMC4138020

[9] Binder D, Drepper T, Jaeger K-E, Delvigne F, Wiechert W, Kohlheyer D, Grünberger A. 2017. Homogenizing bacterial cell factories: analysis and engineering of phenotypic heterogeneity. Metab Eng 42:145–156. doi:10.1016/j.ymben.2017.06.009. 28645641

[10] Delvigne F, Goffin P. 2014. Microbial heterogeneity affects bioprocess robustness: Dynamic single-cell analysis contributes to understanding of microbial populations. Biotechnol J 9:61–72. doi:10.1002/biot.201300119. 24408611

[11] Acar M, Mettetal JT, van Oudenaarden A. 2008. Stochastic switching as a survival strategy in fluctuating environments. Nat Genet 40:471–475. doi:10.1038/ng.110. 18362885

[12] Bishop AL, Rab FA, Sumner ER, Avery SV. 2007. Phenotypic heterogeneity can enhance rare-cell survival in “stress-sensitive” yeast populations. Mol Microbiol 63:507–520. doi:10.1111/j.1365-2958.2006.05504.x. 17176259

[13] Taniguchi Y, Choi PJ, Li G-W, Chen H, Babu M, Hearn J, Emili A, Xie XS. 2010. Quantifying *E. coli* proteome and transcriptome with single- molecule sensitivity in single cells. Science 329:533–538. doi:10.1126/science.1188308. 20671182 PMC2922915

[14] Silander OK, Nikolic N, Zaslaver A, Bren A, Kikoin I, Alon U, Ackermann M. 2012. A genome-wide analysis of promoter-mediated phenotypic noise in *Escherichia coli*. PLoS Genet 8. doi:10.1371/journal.pgen.1002443. PMC3261926 22275871

[15] Newman JRS, Ghaemmaghami S, Ihmels J, Breslow DK, Noble M, DeRisi JL, Weissman JS. 2006. Single-cell proteomic analysis of *S. cerevisiae* reveals the architecture of biological noise. Nature 441:840–846. doi:10.1038/nature04785. 16699522

[16] Kaster AK, Sobol MS. 2020. Microbial single-cell omics: the crux of the matter. Appl Microbiol Biotechnol 104:8209–8220. doi:10.1007/s00253-020-10844-0. 32845367 PMC7471194

[17] Pozsgai ER, Blair KM, Kearns DB. 2012. Modified *mariner* transposons for random inducible-expression insertions and transcriptional reporter fusion insertions in *Bacillus subtilis*. Appl Environ Microbiol 78:778–785. doi:10.1128/AEM.07098-11. 22113911 PMC3264109

[18] Le Breton NP, Mohapatra Y, Haldenwang WG. 2006. In vivo random mutagenesis of *Bacillus subtilis* by use of Tn*YLB-1*, a *mariner*-based transposon. Appl Environ Microbiol 72:327–333. doi:10.1128/AEM.72.1.327-333.2006. 16391061 PMC1352254

[19] Picardeau M. 2010. Transposition of fly *mariner* elements into bacteria as a genetic tool for mutagenesis. Genetica 138:551–558. doi:10.1007/s10709-009-9408-5. 19757097

[20] Overkamp W, Beilharz K, Detert Oude Weme R, Solopova A, Karsens H, Kovács ÁT, Kok J, Kuipers OP, Veening J-W. 2013. Benchmarking various green fluorescent protein variants in *Bacillus subtilis*, *Streptococcus pneumoniae*, and *Lactococcus lactis* for live cell imaging. Appl Environ Microbiol 79:6481–6490. doi:10.1128/AEM.02033-13. 23956387 PMC3811213

[21] Guiziou S, Sauveplane V, Chang H-J, Clerté C, Declerck N, Jules M, Bonnet J. 2016. A part toolbox to tune genetic expression in *Bacillus subtilis*. Nucleic Acids Res 44:7495–7508. doi:10.1093/nar/gkw624. 27402159 PMC5009755

[22] Kunst F, Ogasawara N, Moszer I, Albertini AM, Alloni G, Azevedo V, Bertero MG, Bessières P, Bolotin A, Borchert S, Borriss R, Boursier L, Brans A, Braun M, Brignell SC, Bron S, Brouillet S, Bruschi CV, Caldwell B, Capuano V, Carter NM, Choi S-K, Codani J-J, Connerton IF, Cummings NJ, Daniel RA, Denizot F, Devine KM, Düsterhöft A, Ehrlich SD, Emmerson PT, Entian KD, Errington J, Fabret C, Ferrari E, Foulger D, Fritz C, Fujita M, Fujita Y, Fuma S, Galizzi A, Galleron N, Ghim S-Y, Glaser P, Goffeau A, Golightly EJ, Grandi G, Guiseppi G, Guy BJ, Haga K, et al. 1997. The complete genome sequence of the Gram-positive bacterium *Bacillus subtilis*. Nature 390:249–256. doi:10.1038/36786. 9384377

[23] Markowitz VM, et al. 2012. IMG: the integrated microbial genomes database and comparative analysis system. Nucleic Acids Res 40:115–122. doi:10.1093/nar/gkr1044. PMC3245086 22194640

[24] Cozy LM, Kearns DB. 2010. Gene position in a long operon governs motility development in *Bacillus subtilis*. Mol Microbiol 76:273–285. doi:10.1111/j.1365-2958.2010.07112.x. 20233303 PMC2911795

[25] Kearns DB, Losick R. 2005. Cell population heterogeneity during growth of *Bacillus subtilis*. Genes Dev 19:3083–3094. doi:10.1101/gad.1373905. 16357223 PMC1315410

[26] Chen R, Guttenplan SB, Blair KM, Kearns DB. 2009. Role of the σD-dependent autolysins in *Bacillus subtilis* population heterogeneity. J Bacteriol 191:5775–5784. doi:10.1128/JB.00521-09. 19542270 PMC2737971

[27] Kodama T, Takamatsu H, Asai K, Kobayashi K, Ogasawara N, Watabe K. 1999. The *Bacillus subtilis* yaaH gene is transcribed by SigE RNA polymerase during sporulation, and its product is involved in germination of spores. J Bacteriol 181:4584–4591. doi:10.1128/JB.181.15.4584-4591.1999. 10419957 PMC103590

[28] Imamura D, Kuwana R, Takamatsu H, Watabe K. 2010. Localization of proteins to different layers and regions of *Bacillus subtilis* spore coats. J Bacteriol 192:518–524. doi:10.1128/JB.01103-09. 19933362 PMC2805314

[29] Morales TGP, Ho TD, Liu WT, Dorrestein PC, Ellermeier CD. 2013. Production of the cannibalism toxin SDP is a multistep process that requires SdpA and SdpB. J Bacteriol 195:3244–3251. doi:10.1128/JB.00407-13. 23687264 PMC3697648

[30] González-Pastor JE. 2011. Cannibalism: a social behavior in sporulating *Bacillus subtilis*. FEMS Microbiol Rev 35:415–424. doi:10.1111/j.1574-6976.2010.00253.x. 20955377

[31] de Jong IG, Veening JW, Kuipers OP. 2012. Single cell analysis of gene expression patterns during carbon starvation in *Bacillus subtilis* reveals large phenotypic variation. Environ Microbiol 14:3110–3121. doi:10.1111/j.1462-2920.2012.02892.x. 23033921

[32] Ramírez-Guadiana FH, del Carmen Barajas-Ornelas R, Ayala-García VM, Yasbin RE, Robleto E, Pedraza-Reyes M. 2013. Transcriptional coupling of DNA repair in sporulating *Bacillus subtilis* cells. Mol Microbiol 90:1088–1099. doi:10.1111/mmi.12417. 24118570

[33] Norman TM, Lord ND, Paulsson J, Losick R. 2013. Memory and modularity in cell-fate decision making. Nature 503:481–486. doi:10.1038/nature12804. 24256735 PMC4019345

[34] Syvertsson S, Wang B, Staal J, Gao Y, Kort R, Hamoen LW. 2021. Different resource allocation in a *Bacillus subtilis* population displaying bimodal motility. J Bacteriol 203:e00037-21. doi:10.1128/JB.00037-21. PMC8316123 33782055

[35] Koirala S, Mears P, Sim M, Golding I, Chemla YR, Aldridge PD, Rao CV. 2014. A nutrient-tunable bistable switch controls motility in *Salmonella enterica* serovar Typhimurium. mBio 5:e01611-14. doi:10.1128/mBio.01611-14. 25161191 PMC4173784

[36] Kim JM, Garcia-Alcala M, Balleza E, Cluzel P. 2020. Stochastic transcriptional pulses orchestrate flagellar biosynthesis in Escherichia coli. Sci Adv 6. doi:10.1126/sciadv.aax0947. PMC7002133 32076637

[37] Russell JR, Cabeen MT, Wiggins PA, Paulsson J, Losick R. 2017. Noise in a phosphorelay drives stochastic entry into sporulation in *Bacillus subtilis*. EMBO J 36:2856–2869. doi:10.15252/embj.201796988. 28838935 PMC5623841

[38] Fujita M, Losick R. 2005. Evidence that entry into sporulation in *Bacillus subtilis* is mediated by gradual activation of a master regulator. Genes Dev 19:2236–2244. doi:10.1101/gad.1335705. 16166384 PMC1221893

[39] Errington J. 2003. Regulation of endospore formation in *Bacillus subtilis*. Nat Rev Microbiol 1:117–126. doi:10.1038/nrmicro750. 15035041

[40] Zhu B, Stülke J. 2018. SubtiWiki in 2018: from genes and proteins to functional network annotation of the model organism *Bacillus subtilis*. Nucleic Acids Res 46:D743–D748. doi:10.1093/nar/gkx908. 29788229 PMC5753275

[41] Wang ST, Setlow B, Conlon EM, Lyon JL, Imamura D, Sato T, Setlow P, Losick R, Eichenberger P. 2006. The forespore line of gene expression in *Bacillus subtilis*. J Mol Biol 358:16–37. doi:10.1016/j.jmb.2006.01.059. 16497325

[42] Mars RAT, Nicolas P, Ciccolini M, Reilman E, Reder A, Schaffer M, Mäder U, Völker U, van Dijl JM, Denham EL. 2015. Small regulatory RNA-induced growth rate heterogeneity of *Bacillus subtilis*. PLoS Genet 11:e1005046–27. doi:10.1371/journal.pgen.1005046. 25790031 PMC4366234

[43] Molle V, Fujita M, Jensen ST, Eichenberger P, González-Pastor JE, Liu JS, Losick R. 2003. The Spo0A regulon of *Bacillus subtilis*. Mol Microbiol 50:1683–1701. doi:10.1046/j.1365-2958.2003.03818.x. 14651647

[44] Strauch M, Webb V, Spiegelman G, Hoch JA. 1990. The SpoOA protein of *Bacillus subtilis* is a repressor of the abrB gene. Proc Natl Acad Sci USA 87:1801–1805. doi:10.1073/pnas.87.5.1801. 2106683 PMC53571

[45] Ogura M, Fujita Y. 2007. *Bacillus subtilis* rapD, a direct target of transcription repression by RghR, negatively regulates srfA expression. FEMS Microbiol Lett 268:73–80. doi:10.1111/j.1574-6968.2006.00559.x. 17227471

[46] Smits WK, Eschevins CC, Susanna KA, Bron S, Kuipers OP, Hamoen LW. 2005. Stripping *Bacillus*: ComK auto-stimulation is responsible for the bistable response in competence development. Mol Microbiol 56:604–614. doi:10.1111/j.1365-2958.2005.04488.x. 15819618

[47] Schultz AC, Nygaard P, Saxild HH. 2001. Functional analysis of 14 genes that constitute the purine catabolic pathway in *Bacillus subtilis* and evidence for a novel regulon controlled by the PucR transcription activator. J Bacteriol 183:3293–3302. doi:10.1128/JB.183.11.3293-3302.2001. 11344136 PMC99626

[48] Winstedt L, Yoshida KI, Fujita Y, Wachenfeldt CV. 1998. Cytochrome *bd* biosynthesis in *Bacillus subtilis*: characterization of the cydABCD operon. J Bacteriol 180:6571–6580. doi:10.1128/JB.180.24.6571-6580.1998. 9852001 PMC107760

[49] Ferreira MJ, De Sá-Nogueira I. 2010. A multitask ATPase serving different ABC-type sugar importers in *Bacillus subtilis*. J Bacteriol 192:5312–5318. doi:10.1128/JB.00832-10. 20693325 PMC2950484

[50] Morabbi Heravi K, Watzlawick H, Altenbuchner J. 2019. The melREDCA operon encodes a utilization system for the raffinose family of oligosaccharides in *Bacillus subtilis*. J Bacteriol 201:1–12. doi:10.1128/JB.00109-19. PMC6620409 31138628

[51] Ferreira MJ, Mendes AL, de Sá-Nogueira I. 2017. The MsmX ATPase plays a crucial role in pectin mobilization by *Bacillus subtilis*. PLoS One 12:e0189483–22. doi:10.1371/journal.pone.0189483. 29240795 PMC5730181

[52] Stempler O, et al. 2017. Interspecies nutrient extraction and toxin delivery between bacteria. Nat Commun 8:315. doi:10.1038/s41467-017-00344-7. 28827522 PMC5566331

[53] Su Y, Liu C, Fang H, Zhang D. 2020. *Bacillus subtilis*: a universal cell factory for industry, agriculture, biomaterials and medicine. Microb Cell Fact 19:173. doi:10.1186/s12934-020-01436-8. 32883293 PMC7650271

[54] Liu Y, Liu L, Li J, Du G, Chen J. 2019. Synthetic biology toolbox and chassis development in *Bacillus subtilis*. Trends Biotechnol 37:548–562. doi:10.1016/j.tibtech.2018.10.005. 30446263

[55] De Jong IG, Beilharz K, Kuipers OP, Veening J-W. 2011. Live cell imaging of *Bacillus subtilis* and *Streptococcus pneumoniae* using automated time-lapse microscopy. J Vis Exp 53. doi:10.3791/3145. PMC3197447 21841760

[56] Kort R, O'Brien AC, van Stokkum IHM, Oomes SJCM, Crielaard W, Hellingwerf KJ, Brul S. 2005. Assessment of heat resistance of bacterial spores from food product isolates by fluorescence monitoring of dipicolinic acid release. Appl Environ Microbiol 71:3556–3564. doi:10.1128/AEM.71.7.3556-3564.2005. 16000762 PMC1169001

[57] Chan JM, Guttenplan SB, Kearns DB. 2014. Defects in the flagellar motor increase synthesis of poly-γ-glutamate in bacillus subtilis. J Bacteriol 196:740–753. doi:10.1128/JB.01217-13. 24296669 PMC3911173

