# Supplementary material for: High-Throughput Time-Lapse Fluorescence Microscopy Screening for Heterogeneously Expressed Genes in Bacillus subtilis
Source: Microbiol Spectr. 2022 Feb 16;10(1):e02045-21. doi: 10.1128/spectrum.02045-21 (PMC8849057; doi:10.1128/spectrum.02045-21)
Supplement: SUPPLEMENTAL FILE 1 — Supplemental material. Download SPECTRUM02045-21_Supp_1_seq8.pdf, PDF file, 0.5 MB [file spectrum02045-21_supp_1_seq8.pdf]

Supplementary Table S1. Overview of strains used in this study

Strain	Description	Source or reference
Escherichia coli		
DH5α	Used for transformation of plasmids	Laboratory collection
Bacillus subtilis		
PS832	*B. subtilis* PS832 wild-type; prototrophic derivative of *B. subtilis* 168	Received from Peter Setlow (University of Connecticut)
PS832 pKB176-TnJM1	PS832 harboring the pKB176 delivery vector for transposition of TnJM1	This study
PS832 *albA::TnJM1*[1]	PS832 with the TnJM1 transposon inserted into *albA*	This study
PS832 *cydB::TnJM1*[1]	PS832 with the TnJM1 transposon inserted into *cydB*	This study
PS832 *cypX::TnJM1*[1]	PS832 with the TnJM1 transposon inserted into *cypX*	This study
PS832 *dhbB::TnJM1* (1)[1]	PS832 with the TnJM1 transposon inserted into *dhbB*	This study
PS832 *dhbB::TnJM1* (2)[1]	PS832 with the TnJM1 transposon inserted into *dhbB*	This study
PS832 *dhbE::TnJM1*[1]	PS832 with the TnJM1 transposon inserted into *dhbE*	This study
PS832 *dhbF::TnJM1*[1]	PS832 with the TnJM1 transposon inserted into *dhbF*	This study
PS832 *epeE::TnJM1*[1]	PS832 with the TnJM1 transposon inserted into *epeE*	This study
PS832 *flgK::TnJM1*[1]	PS832 with the TnJM1 transposon inserted into *flgK*	This study
PS832 *hag::TnJM1* (1)[1]	PS832 with the TnJM1 transposon inserted into *hag*	This study
PS832 *hag::TnJM1* (2)[1]	PS832 with the TnJM1 transposon inserted into *hag*	This study
PS832 *lytA::TnJM1*[1]	PS832 with the TnJM1 transposon inserted into *lytA*	This study
PS832 *lytF::TnJM1*[1]	PS832 with the TnJM1 transposon inserted into *lytF*	This study
PS832 *mfd::TnJM1*[1]	PS832 with the TnJM1 transposon inserted into *mfd*	This study
PS832 *msmX::TnJM1*[1]	PS832 with the TnJM1 transposon inserted into *msmX*	This study
PS832 *pucF::TnJM1*[1]	PS832 with the TnJM1 transposon inserted into *pucF*	This study
PS832 *rapD::TnJM1*[1]	PS832 with the TnJM1 transposon inserted into *rapD*	This study
PS832 *sdpB::TnJM1*[1]	PS832 with the TnJM1 transposon inserted into *sdpB*	This study
PS832 *wapA::TnJM1*[1]	PS832 with the TnJM1 transposon inserted into *wapA*	This study
PS832 *yaaH::TnJM1*[1]	PS832 with the TnJM1 transposon inserted into *yaaH*	This study
PS832 *yfmG::TnJM1*[1]	PS832 with the TnJM1 transposon inserted into *yfmG*	This study

[1] *sfGFP(Sp)* from the TnJM1 transposon is inserted in the same orientation as the gene it is inserted into.

Supplementary Table S2. Overview of plasmids used in this study.

Plasmid	Description	Source or reference
pDR111-sfgfp(Sp)	Contains *sfgfp(Sp)*	[1]
pKB157	Contains a *mariner* transposon with a kanamycin resistance gene	[2]
pKB157-TnJM1	pKB157 with *sfgfp(Sp)* inserted into the *mariner* transposon	This study
pKB176	Delivery vector that encodes the Himar1 transposase	[2]
pKB176-TnJM1	pKB176 containing the TnJM1 transposon	This study

Supplementary Table S3. Overview of primers used in this work. When relevant, primer attachment sites are indicated in bold and restriction sites in red.

Name	Sequence	Purpose
P1	ACGCGTCGACTAAGTGAG**GATTAACTAATAAGG AGGACAAACATGTC**	Amplification of *sfgfp(Sp)* for insertion in pKB157; contains a SalI restriction site
P2	CGCGGATCC**TTATTTATAAAGTTCGTCCATACC GTGAG**	Amplification of *sfgfp(Sp)* for insertion in pKB157; contains a BamHI restriction site
P3	CGCCACCTCTGACTTGAG	PCR verification and sequencing of *sfgfp(Sp)* insertion in pKB157
P4	GGCGTATAACATAGTATCGACGGAG	PCR verification and sequencing of *sfgfp(Sp)* insertion in pKB157
P5	GCGGGCCTCTTCGCTATTAC	Sequencing of *sfgfp(Sp)* insertion in pKB157
P6	CAATCAGGCTTGATCCCCAG	Sequencing of *sfgfp(Sp)* insertion in pKB157
P7	GCTCATGAGTGAGGCCGATG	Sequencing of *sfgfp(Sp)* insertion in pKB157
P8	GCGTCGCTAGTATTAAATGC	Sequencing of *sfgfp(Sp)* insertion in pKB157
P9	GTGAGCGGATAACAATTTCACA	Amplification of TnJM1 for insertion in pKB176
P10	GGCCAGTGAATTCGAGCT	Amplification of TnJM1 for insertion in pKB176
P11	GTATCGCTCTTGAAGGGAACTATG	PCR verification and sequencing of TnJM1 insertion in pKB176
P12	GTGGATAACCGTATTACCGCC	PCR verification and sequencing of TnJM1 insertion in pKB176
P13	GTTAATCCTCACTTAGTCGACC	Primer of Y-linker protocol that attaches to TnJM1
P14	CTGCTCGAATTCAAGCTTCT	Primer of Y-linker protocol that becomes ligated to NlaIII digested genomic fragments
P15	TTTCTGCTCGAATTCAAGCTTCTAACGATGTACG GGGACACATG	Construction of Y-linker.
P16	TGTCCCCGTACATCGTTAGAACTACTCGTACCATC CACAT	Construction of Y-linker. 5' is phosphorylated.
P17	GTGCAACAGGTCTATTCGGTC	PCR verification and sequencing of *albA* locus
P18	GGTTCACGCCTGTCAGC	PCR verification and sequencing of *albA* locus
P19	CGGTGTGATGTACATGATTCTTG	PCR verification and sequencing of *cydB* locus
P20	CAGCGTTGCATGATAATCATTCC	PCR verification and sequencing of *cydB* locus
P21	CATCTGAAGGATGATAGGGG	PCR verification and sequencing of *cypX* locus
P22	GTAAAAAGAGTCTCACCCGC	PCR verification and sequencing of *cypX* locus
P23	CAGGCATCCGTTTGTTTACC	PCR verification and sequencing of *dhbB* locus
P24	CGCAGACAGGAGTAGGAAAA	PCR verification and sequencing of *dhbB* locus
P25	CGGCTGAATGGCAGGTATAG	PCR verification and sequencing of *dhbE* locus
P26	ATGAATTGCTGGAGGGATAC	PCR verification and sequencing of *dhbE* locus
P27	CTACAAGTCTCTCAAACGGC	PCR verification and sequencing of *dhbF* locus
P28	ATGCTGGAGGATGCGAAACC	PCR verification and sequencing of *dhbF* locus
P29	GAGTCTAACTCTCCTTTACC	PCR verification and sequencing of *epeE* locus
P30	CGTTGTTTTGCACAAGCACTTC	PCR verification and sequencing of epeE locus
P31	GCCTTGTGTTACTCTCATGT	PCR verification and sequencing of *flgK* locus
P32	GACATCTACCTTTATGGGGC	PCR verification and sequencing of *flgK* locus
P33	GTGATCTCCGCATTATCCTCAC	PCR verification and sequencing of *hag* locus
P34	GAGTTAGTGACAGGATGAGGAATG	PCR verification and sequencing of *hag* locus
P35	GCGTTCATCCACACCCATG	PCR verification and sequencing of *lytA* locus
P36	CAATTTAACTGAGATGTTTGAGTCTGCTG	PCR verification and sequencing of *lytA* locus
P37	GCATCAACGTCTTTAGGCTC	PCR verification and sequencing of *lytF* locus
P38	ACGATGAGACAAATGTGCCG	PCR verification and sequencing of *lytF* locus
P39	TGTTCTCGCAGGAAACAGAG	PCR verification and sequencing of *mfd* locus
P40	TTATGTACCTTCCGCCTCGC	PCR verification and sequencing of *mfd* locus
P41	GCTGAATTGCGGATGGAGC	PCR verification and sequencing of *msmX* locus
P42	GACCTTTATTCATATCAAATGCGACCG	PCR verification and sequencing of *msmX* locus
P43	CCCATTGCCTTTATCCCTGC	PCR verification and sequencing of *pucF* locus
P44	CATGTTGCTCCTTCTATAATGAAGTCAC	PCR verification and sequencing of *pucF* locus

P45	GTATAGCAATCATCAAAGATCCAATCTGTAC	PCR verification and sequencing of *rapD* locus
P46	CAATACAGTGAACATCGACAGCTCAATG	PCR verification and sequencing of *rapD* locus
P47	GGTTGAGCAGGACTACTATCAAAC	PCR verification and sequencing of *sdpB* locus
P48	CCAATATCGTTACCATGGAAACAATC	PCR verification and sequencing of *sdpB* locus
P49	CATACGTAGCCCATTCCTATTATGG	PCR verification and sequencing of *wapA* locus
P50	GCATCACCAACATCAAAGGACTG	PCR verification and sequencing of *wapA* locus
P51	CGATTCCTAAGGACTGTATCGCG	PCR verification and sequencing of *yaaH* locus
P52	CGTGCGTCAGTTACCGACC	PCR verification and sequencing of *yaaH* locus
P53	CGGGAGGTTATCGTAATTTG	PCR verification and sequencing of *yfmG* locus
P54	GTGACAGCCTTCATTTTCCC	PCR verification and sequencing of *yfmG* locus

Supplementary Information S1. Sequences of TnJM1 insertion sites of the reconstructed mutants. Sequence corresponding to the TnJM1 transposon is indicated in red.

*albA::*TnJM1

TCGGGATCTCGGGGCGAAGGCGTTTTCTTACAATTGGGTTGACGATTTCGGAAGAGGCAGGGATATTGTCCATCCAACGAAAG
ACGCCGAGCAGCACCGCAAGTTTATGGAATACGAGCAACATGTGATTGATGAGTTTAAAGATCTGATTCCGATTATTCCCTAT
GAGAGAAAACGCGCGGCAAATTGCGGCGCTGGCTGGAAGTCCATTGTGATCAGTCCGTTCGGCGAAGTACGTCCTTGCGCCCTC
TTTCCAAAGGAATTTTCATTGGGAAATATTTTTCATGATTCCTATGAAAGCATCTTTAACTCCCCTCTCGTCCATAAACTGTG
GCAAGCGCAAGCGCCGCGGTTCAGCGAACATTGCATGAAAGACAAATGCCCGTTCAGCGGCTATTGCGGAGGCTGTTACTTAA
AAGGGCTGAACTCTAACAAATATCACCGGAAAAACATTTGCTCTTGGGCGAAAAATGAACAATTAGAAGACGTGGTCCAGCTT
AACAGGTTGGCTGATAAGTCCCCGGTCTGGTCGACTAAGTGAGGATTAACTAATAAGGAGGACAAACATGTCAAAAGGAGAA
GAACTTTTTACAGGTGTAGTACCTATCTTGGTTGAATTGGATGGTGATGTTAACGGTCACAAATTTTCTGTACGTGGTGAAGG
TGAAGGTGATGCAACTAACGGTAAATTGACACTTAAATTCATTTGTACAACTGGAAAACTTCCTGTTCCTTGGCCTACTCTTG
TTACAACATTGACATATGGAGTACAATGTTTTTCACGTTATCCTGATCATATGAAACGTCACGATTTTTTTAAATCTGCTATG
CCAGAAGGTTATGTACAAGAACGTACAATTTCATTTAAAGATGACGGAACATATAAAACACGTGCTGAAGTAAAATTCGAAG
GTGACACTCTTGTTAATCGTATCGAATTGAAAGGAATCGATTTCAAAGAAGATGGTAACATTTTGGGACACAAACTTGAATAC
AACTTCAACTCTCATAATGTTTATATCACAGCTGACAAACAAAAAAACGGTATTAAAGCTAATTTTAAAATTCGTCACAATGT
TGAAGATGGATCTGTTCAATTGGCTGATCATTATCAACAAAATACACCAATCGGAGACGGACCAGTATTGCTTCCAGATAACC
ACTACCTTTCTACTCAATCAGTTCTTTCAAAAGATCCTAACGAAAAACGTGACCATATGGTACTTCTTGAATTTGTTACAGCA
GCAGGTATCACTCACGGTATGGACGAACTTTATAAATAAGGATCCTACTCGAGCCACATAGATGGCGTCGCTAGTATTAAATG
CATATTATTTTTATATAGTACCAACCTTCAAATGATTCCCTATAGCTTGTAAATTCTATCATAATTGTGGTTTCAAAATCGGC
TCCGTCGATACTATGTTATACGCCAACTTTGAAAACAACTTTGAAAAAGCTGTTTTCTGGTATTTAAGGTTTTAGAATGCAAG
GAACAGTGAATTGGAGTTCGTCTTGTTATAATTAGCTTCTTGGGGTATCTTTAAATACTGTAGAAAAGAGGAAGGAAATAAT
AAATGGCTAAAATGAGAATATCACCGGAATTGAAAAAACTGATCGAAAAATACCGCTGCGTAAAAGATACGGAAGGAATGTC
TCCTGCTAAGGTATATAAGCTGGTGGGAGAAAATGAAAACCTATATTTAAAAATGACGGACAGCCGGTATAAAGGGACCACCT
ATGATGTAGAACGGGAAAAGGACATGATGCTATGGCTGGAAGGAAAGCTGCCTGTTCCAAAGGTCCTGCACTTTGAACGGCAT
GATGGCTGGAGCAATCTGCTCATGAGTGAGGCCGATGGCGTCCTTTGCTCGGAAGAGTATGAAGATGAACAAAGCCCTGAAAA
GATTATCGAGCTGTATGCGGAGTGCATCAGGCTCTTTCACTCCATCGACATATCGGATTGTCCCTATACGAATAGCTTAGACA
GCCGCTTAGCCGAATTGGATTACTTACTGAATAACGATCTGGCCGATGTGGATTGCGAAAACTGGGAAGAAGACACTCCATTT
AAAGATCCGCGCGAGCTGTATGATTTTTTAAAGACGGAAAAGCCCGAAGAGGAACTTGTCTTTTCCCACGGCGACCTGGGAGA
CAGCAACATCTTTGTGAAAGATGGCAAAGTAAGTGGCTTTATTGATCTTGGGAGAAGCGGCAGGGCGGACAAGTGGTATGACA
TTGCCTTCTGCGTCCGGTCGATCAGGGAGGATATCGGGGAAGAACAGTATGTCGAGCTATTTTTTGACTTACTGGGGATCAAG
CCTGATTGGGAGAAAATAAAATATTATATTTTACTGGATGAATTGTTTTAGTACCTAGATTTAGATGTCTAAAGAATTGATG
CTTTAACTACATGCTTTTTAGACATCTAATCTTTTCTGAAGTACATCCGCAACTGTCCATACTCTGATGTTTTATATCTTTTCT
AAAAGTTCGCTAGATAGGGGTCCCGAGCGCCTACGAGGAATTTGTATCGGGGTATAGATCTAAAACGAATTTTAACAAAAAAA
TGTGTTTTTCTTTGTTCTCGAGCCCGGGGCTAGCACTAGTAGACCGGGGACTTATCAGCCAACCTGTTTTAGGAGGGAAACGA
TTTGTCACCAGCACAAAGAAGAATTTTACTGTATATCCTTTCATTTATCTTTGTCATCGGCGCAGTCGTCTATTTTGTCAAAA
GCGATTATCTGTTTACGCTGATTTTCATTGCCATTGCCATTCTGTTCGGGATGCGCGCGCGGAAGGCTGACTCGCGATGAGCAT
TTTGGATATACACGATGTATCCGTTTGGTATGAACGGGACAACGTCATCTTAGAACAAGTGGATTTACATTTAGAAAAAGGCG
CCGTTTACGGGTTACTTGGGGTAAACGGCGCCGGAAAAACGACACTGATTAATACGCTGACAGGCGTGAACC

*cydB::*TnJM1

TAATTCCTCCGTTTGTGCTTGGTGTGCTGTTCACGACATTATTCCGCGGGATGCCGATTGATGCCGACATGAACATTCACGCAC
ATGTATCTGATTATATCAATGTATATTCTATACTTGGCGGTGTGACGGTAACGCTTCTTTGCTTCCAGCATGGATTAATGTTT
ATCACGCTGCGTACGATTGGTGATTTGCAAAACCGCGCCCGGAAAATGGCGCAAAAGATAATGGGTGTTGTTTTTGTAGCGGT
TCTTGCCTTCGCGGCCCTTTCTGCGTATCAGACAGACATGTTTACACGCCGCGGCGAGATTACCATTCCGCTCGCTGTTTTGAT
TGTCATCTGCTTTATGCTCGCGGCTGTATTTATTCGTAAGAAAAAAGACGGCTGGACATTTGGGATGACAGGCGCTGGTCTGG
CGCTGACCGTGGGAATGATCTTTATTTCCCTATTCCCTCGTGTAATGGTCAGCTCGCTTCATAGCGCATACGATTTAACAGTAA
CAGGTTGGCTGATAAGTCCCCGGTCTGGTCGACTAAGTGAGGATTAACTAATAAGGAGGACAAACATGTCAAAAGGAGAAGA
ACTTTTTACAGGTGTAGTACCTATCTTGGTTGAATTGGATGGTGATGTTAACGGTCACAAATTTTCTGTACGTGGTGAAGGTG
AAGGTGATGCAACTAACGGTAAATTGACACTTAAATTCATTTGTACAACTGGAAAACTTCCTGTTCCTTGGCCTACTCTTGTT
ACAACATTGACATATGGAGTACAATGTTTTTCACGTTATCCTGATCATATGAAACGTCACGATTTTTTTAAATCTGCTATGCC
AGAAGGTTATGTACAAGAACGTACAATTTCATTTAAAGATGACGGAACATATAAAACACGTGCTGAAGTAAAATTCGAAGGT
GACACTCTTGTTAATCGTATCGAATTGAAAGGAATCGATTTCAAAGAAGATGGTAACATTTTGGGACACAAACTTGAATACAA
CTTCAACTCTCATAATGTTTATATCACAGCTGACAAACAAAAAAACGGTATTAAAGCTAATTTTAAAATTCGTCACAATGTTG
AAGATGGATCTGTTCAATTGGCTGATCATTATCAACAAAATACACCAATCGGAGACGGACCAGTATTGCTTCCAGATAACCAC
TACCTTTCTACTCAATCAGTTCTTTCAAAAGATCCTAACGAAAAACGTGACCATATGGTACTTCTTGAATTTGTTACAGCAGC
AGGTATCACTCACGGTATGGACGAACTTTATAAATAAGGATCCTACTCGAGCCACATAGATGGCGTCGCTAGTATTAAATGCA
TATTATTTTTATATAGTACCAACCTTCAAATGATTCCCTATAGCTTGTAAATTCTATCATAATTGTGGTTTCAAAATCGGCTC

CGTCGATACTATGTTATACGCCAACTTTGAAAACAACTTTGAAAAAGCTGTTTTCTGGTATTTAAGGTTTTAGAATGCAAGGA
ACAGTGAATTGGAGTTCGTCTTGTTATAATTAGCTTCTTGGGGTATCTTTAAATACTGTAGAAAAGAGGAAGGAAATAATAA
ATGGCTAAAATGAGAATATCACCGGAATTGAAAAAACTGATCGAAAAATACCGCTGCGTAAAAGATACGGAAGGAATGTCTC
CTGCTAAGGTATATAAGCTGGTGGGAGAAAATGAAAACCTATATTTAAAAATGACGGACAGCCGGTATAAAGGGACCACCTAT
GATGTAGAACGGGAAAAGGACATGATGCTATGGCTGGAAGGAAAGCTGCCTGTTCCAAAGGTCCTGCACTTTGAACGGCATGA
TGGCTGGAGCAATCTGCTCATGAGTGAGGCCGATGGCGTCCTTTGCTCGGAAGAGTATGAAGATGAACAAAGCCCTGAAAAGA
TTATCGAGCTGTATGCGGAGTGCATCAGGCTCTTTCACTCCATCGACATATCGGATTGTCCCTATACGAATAGCTTAGACAGCC
GCTTAGCCGAATTGGATTACTTACTGAATAACGATCTGGCCGATGTGGATTGCGAAAACTGGGAAGAAGACACTCCATTTAAA
GATCCGCGCGAGCTGTATGATTTTTTAAAGACGGAAAAGCCCGAAGAGGAACTTGTCTTTTCCCACGGCGACCTGGGAGACAG
CAACATCTTTGTGAAAGATGGCAAAGTAAGTGGCTTTATTGATCTTGGGAGAAGCGGCAGGGCGGACAAGTGGTATGACATTG
CCTTCTGCGTCCGGTCGATCAGGGAGGATATCGGGGAAGAACAGTATGTCGAGCTATTTTTTGACTTACTGGGGATCAAGCCT
GATTGGGAGAAAATAAAATATTATATTTTACTGGATGAATTGTTTTAGTACCTAGATTTAGATGTCTAAAGAATTGATGCTT
TAACTACATGCTTTTTAGACATCTAATCTTTTCTGAAGTACATCCGCAACTGTCCATACTCTGATGTTTTATATCTTTTCTAAA
AGTTCGCTAGATAGGGGTCCCGAGCGCCTACGAGGAATTTGTATCGGGGTATAGATCTAAAACGAATTTTAACAAAAAAATGT
GTTTTTCTTTGTTCTCGAGCCCGGGGCTAGCACTAGTAGACCGGGGACTTATCAGCCAACCTGTTAGCTAACGCTTCTTCTGGA
GATTATTCGCTTAAAGTCATGTCAATTGCAGCATTAACGCTATTGCCGTTCGTCATCGGCAGCCAGATCTGGAGCTATTATGT
CTTCCGGAAGCGCGTCAGCCATAAGGAGCCTATGACTTATTAATGGGAAAAGACCTGTTTCGATATAAAGGAATGAAGCGGAT
TCTCACCTTGATTACTTGTTTAACGTTGATTCAGACAGCCGCCATTATTATGCAGGCAGAGTGGCTGAGTGAGGCCGTAACCG
GACTGTTCAACGGGAAGGGCATTACCTCCCTTTTACCGGTGATCGGCTTTTTCCTCATCGCTTTTATCGCCCCGGCACGGATGA
CGGTGGCGCGACAGAAAATCGTCTATCAATACGCCGCCCGGACAGGAGCAGATTTGAGGAAAAGTTTCTTGATCAGCTGTTC
CGATTAGGCCCCCGCTTTGCAAAGAAAGAGGGGACGGGGCAAATGGTGACGCTGGCGATGGAAGGCATCAGCCAGTTCCGCCG
CTACCTGGAGCTGTTTC

*cypX::*TnJM1

ATATACTCGCACTGATTCTTAATGTGCTGTTAGCCGCAACGGAACCGGCTGATAAGACGCTGGCACTGATGATCTACCATTTGC
TCAACAATCCTGAGCAGATGAATGATGTTTTGGCTGACCGTTCGTTAGTTCCGAGAGCCATTGCGGAGACATTGCGTTATAAA
CCGCCGGTTCAGCTGATTCCGCGGCAGCTGTCCCAAGATACAGTGGTCGGCGGTATGGAAATCAAAAAAGATACGATTGTTTT
TTGTATGATCGGTGCGGCTAACCGGGACCCTGAAGCATTTGAACAGCCTGACGTGTTTAATATTCATCGGGAAGATCTTGGTA
TCAAGAGCGCTTTTAGCGGCGCCGCCCGGCATCTCGCTTTCGGATCCGGCATTCATAACTGTGTAGGAGCAGCTTTTGCCAAAA
ACGAAATCGAAATTGTAGCTAATATTGTGCTGGATAAGATGCGGAATATCAGATTAGAGGAAGATTTTTGTTATGCTGAGTCC
GGTCTGACAGGTTGGCTGATAAGTCCCCGGTCTGGTCGACTAAGTGAGGATTAACTAATAAGGAGGACAAACATGTCAAAAGG
AGAAGAACTTTTTACAGGTGTAGTACCTATCTTGGTTGAATTGGATGGTGATGTTAACGGTCACAAATTTTCTGTACGTGGTG
AAGGTGAAGGTGATGCAACTAACGGTAAATTGACACTTAAATTCATTTGTACAACTGGAAAACTTCCTGTTCCTTGGCCTACT
CTTGTTACAACATTGACATATGGAGTACAATGTTTTTCACGTTATCCTGATCATATGAAACGTCACGATTTTTTTAAATCTGC
TATGCCAGAAGGTTATGTACAAGAACGTACAATTTCATTTAAAGATGACGGAACATATAAAACACGTGCTGAAGTAAAATTC
GAAGGTGACACTCTTGTTAATCGTATCGAATTGAAAGGAATCGATTTCAAAGAAGATGGTAACATTTTGGGACACAAACTTGA
ATACAACTTCAACTCTCATAATGTTTATATCACAGCTGACAAACAAAAAAACGGTATTAAAGCTAATTTTAAAATTCGTCACA
ATGTTGAAGATGGATCTGTTCAATTGGCTGATCATTATCAACAAAATACACCAATCGGAGACGGACCAGTATTGCTTCCAGAT
AACCACTACCTTTCTACTCAATCAGTTCTTTCAAAAGATCCTAACGAAAAACGTGACCATATGGTACTTCTTGAATTTGTTAC
AGCAGCAGGTATCACTCACGGTATGGACGAACTTTATAAATAAGGATCCTACTCGAGCCACATAGATGGCGTCGCTAGTATTA
AATGCATATTATTTTTATATAGTACCAACCTTCAAATGATTCCCTATAGCTTGTAAATTCTATCATAATTGTGGTTTCAAAAT
CGGCTCCGTCGATACTATGTTATACGCCAACTTTGAAAACAACTTTGAAAAAGCTGTTTTCTGGTATTTAAGGTTTTAGAATG
CAAGGAACAGTGAATTGGAGTTCGTCTTGTTATAATTAGCTTCTTGGGGTATCTTTAAATACTGTAGAAAAGAGGAAGGAAA
TAATAAATGGCTAAAATGAGAATATCACCGGAATTGAAAAAACTGATCGAAAAATACCGCTGCGTAAAAGATACGGAAGGAA
TGTCTCCTGCTAAGGTATATAAGCTGGTGGGAGAAAATGAAAACCTATATTTAAAAATGACGGACAGCCGGTATAAAGGGACC
ACCTATGATGTAGAACGGGAAAAGGACATGATGCTATGGCTGGAAGGAAAGCTGCCTGTTCCAAAGGTCCTGCACTTTGAACG
GCATGATGGCTGGAGCAATCTGCTCATGAGTGAGGCCGATGGCGTCCTTTGCTCGGAAGAGTATGAAGATGAACAAAGCCCTG
AAAAGATTATCGAGCTGTATGCGGAGTGCATCAGGCTCTTTCACTCCATCGACATATCGGATTGTCCCTATACGAATAGCTTA
GACAGCCGCTTAGCCGAATTGGATTACTTACTGAATAACGATCTGGCCGATGTGGATTGCGAAAACTGGGAAGAAGACACTCC
ATTTAAAGATCCGCGCGAGCTGTATGATTTTTTAAAGACGGAAAAGCCCGAAGAGGAACTTGTCTTTTCCCACGGCGACCTGG
GAGACAGCAACATCTTTGTGAAAGATGGCAAAGTAAGTGGCTTTATTGATCTTGGGAGAAGCGGCAGGGCGGACAAGTGGTAT
GACATTGCCTTCTGCGTCCGGTCGATCAGGGAGGATATCGGGGAAGAACAGTATGTCGAGCTATTTTTTGACTTACTGGGGAT
CAAGCCTGATTGGGAGAAAATAAAATATTATATTTTACTGGATGAATTGTTTTAGTACCTAGATTTAGATGTCTAAAGAATT
GATGCTTTAACTACATGCTTTTTAGACATCTAATCTTTTCTGAAGTACATCCGCAACTGTCCATACTCTGATGTTTTATATCTT
TTCTAAAAGTTCGCTAGATAGGGGTCCCGAGCGCCTACGAGGAATTTGTATCGGGGTATAGATCTAAAACGAATTTTAACAAA
AAAATGTGTTTTTCTTTGTTCTCGAGCCCGGGGCTAGCACTAGTAGACCGGGGACTTATCAGCCAACCTGTTATACACGCGGAC
CTGTTTCACTTCTCGTTGCGTTTGACGGGGCATAATAGAATTCCAAAGGTCTCTCCCATGCGGGTGAGACTCTTTTTAC

*dhbB::*TnJM1 (1)

CGGCTGAAGAGGTAGAAAATCATCTGCTGGCGCATCCGGCTGTCCATGATGCGGCAATGGTCTCCATGCCTGATCAATTTCTTG
GCGAAAGATCTTGTGTGTTCATTATTCCCCGGGATGAAGCCCCAAAAGCCGCAGAGCTTAAAGCATTTTTGAGAGAGCGCGGA
CTGGCGGCATATAAAATCCCTGATCGAGTTGAATTTGTCGAATCCTTCCCGCAGACAGGAGTAGGAAAAGTCAGCAAAAAAGC
GCTCCGTGAAGCCATTTCCGAGAAGCTTCTTGCAGGATTTAAAAAATAAAACAACAATTTGAGAGGAAGTGTTCATATGGCTA
TACCTGCCATTCAGCCGTATCAAATGCCGACAGCATCTGATATGCCGCAAAACAAAGTATCATGGGTGCCTGATCCGAATCGG
GCTGTCTTGTTAATACACGATAACAGGTTGGCTGATAAGTCCCCGGTCTGGTCGACTAAGTGAGGATTAACTAATAAGGAGGA
CAAACATGTCAAAAGGAGAAGAACTTTTTACAGGTGTAGTACCTATCTTGGTTGAATTGGATGGTGATGTTAACGGTCACAAA
TTTTCTGTACGTGGTGAAGGTGAAGGTGATGCAACTAACGGTAAATTGACACTTAAATTCATTTGTACAACTGGAAAACTTCC
TGTTCCTTGGCCTACTCTTGTTACAACATTGACATATGGAGTACAATGTTTTTCACGTTATCCTGATCATATGAAACGTCACG
ATTTTTTTAAATCTGCTATGCCAGAAGGTTATGTACAAGAACGTACAATTTCATTTAAAGATGACGGAACATATAAAACACGT
GCTGAAGTAAAATTCGAAGGTGACACTCTTGTTAATCGTATCGAATTGAAAGGAATCGATTTCAAAGAAGATGGTAACATTTT
GGGACACAAACTTGAATACAACTTCAACTCTCATAATGTTTATATCACAGCTGACAAACAAAAAAACGGTATTAAAGCTAATT
TTAAAATTCGTCACAATGTTGAAGATGGATCTGTTCAATTGGCTGATCATTATCAACAAAATACACCAATCGGAGACGGACCA
GTATTGCTTCCAGATAACCACTACCTTTCTACTCAATCAGTTCTTTCAAAAGATCCTAACGAAAAACGTGACCATATGGTACT
TCTTGAATTTGTTACAGCAGCAGGTATCACTCACGGTATGGACGAACTTTATAAATAAGGATCCTACTCGAGCCACATAGATG
GCGTCGCTAGTATTAAATGCATATTATTTTTATATAGTACCAACCTTCAAATGATTCCCTATAGCTTGTAAATTCTATCATAA
TTGTGGTTTCAAAATCGGCTCCGTCGATACTATGTTATACGCCAACTTTGAAAACAACTTTGAAAAAGCTGTTTTCTGGTATT
TAAGGTTTTAGAATGCAAGGAACAGTGAATTGGAGTTCGTCTTGTTATAATTAGCTTCTTGGGGTATCTTTAAATACTGTAGA
AAAGAGGAAGGAAATAATAAATGGCTAAAATGAGAATATCACCGGAATTGAAAAAACTGATCGAAAAATACCGCTGCGTAAA
AGATACGGAAGGAATGTCTCCTGCTAAGGTATATAAGCTGGTGGGAGAAAATGAAAACCTATATTTAAAAATGACGGACAGC
CGGTATAAAGGGACCACCTATGATGTAGAACGGGAAAAGGACATGATGCTATGGCTGGAAGGAAAGCTGCCTGTTCCAAAGGT
CCTGCACTTTGAACGGCATGATGGCTGGAGCAATCTGCTCATGAGTGAGGCCGATGGCGTCCTTTGCTCGGAAGAGTATGAAG
ATGAACAAAGCCCTGAAAAGATTATCGAGCTGTATGCGGAGTGCATCAGGCTCTTTCACTCCATCGACATATCGGATTGTCCC
TATACGAATAGCTTAGACAGCCGCTTAGCCGAATTGGATTACTTACTGAATAACGATCTGGCCGATGTGGATTGCGAAAACTG
GGAAGAAGACACTCCATTTAAAGATCCGCGCGAGCTGTATGATTTTTTAAAGACGGAAAAGCCCGAAGAGGAACTTGTCTTTT
CCCACGGCGACCTGGGAGACAGCAACATCTTTGTGAAAGATGGCAAAGTAAGTGGCTTTATTGATCTTGGGAGAAGCGGCAGG
GCGGACAAGTGGTATGACATTGCCTTCTGCGTCCGGTCGATCAGGGAGGATATCGGGGAAGAACAGTATGTCGAGCTATTTTT
TGACTTACTGGGGATCAAGCCTGATTGGGAGAAAATAAAATATTATATTTTACTGGATGAATTGTTTTAGTACCTAGATTTAG
ATGTCTAAAGAATTGATGCTTTAACTACATGCTTTTTAGACATCTAATCTTTTCTGAAGTACATCCGCAACTGTCCATACTCT
GATGTTTTATATCTTTTCTAAAAGTTCGCTAGATAGGGGTCCCGAGCGCCTACGAGGAATTTGTATCGGGGTATAGATCTAAA
ACGAATTTTAACAAAAAAATGTGTTTTTCTTTGTTCTCGAGCCCGGGGCTAGCACTAGTAGACCGGGGACTTATCAGCCAACC
TGTTATGCAAAACTATTTTGTTGATGCTTTCACAGCGGGAGCGTCTCCGGTAACAGAGCTTTCAGCGAATATACGAAAGCTGA
AGAATCAATGTGTTCAGCTTGGGATTCCTGTTGTCTATACCGCACAGCCGGGAAGCCAAAATCCGGATGACCGTGCGCTGCTG
ACAGACTTTTGGGGCCCGGGATTAAACAGCGGTCCTTATGAGGAGAAAATTATAACCGAGCTGGCACCAGAGGATGATGATCT
TGTGCTGACAAAATGGAGATACAGCGCGTTTAAGAGAACGAATCTGCTTGAAATGATGCGCAAAGAGGGACGCGATCAGCTGA
TCATTACAGGAATTTACGCCCATATCGGCTGTCTTGTTACAGCATGTGAAGCATTTATGGAGGATATTAAAGCCTTTTTTGTG
GGAGATGCAGTTGCTGATTTTTCATTAGAAAAACATCAAATGGCGCTGGAATATGCGGCTGGACGCTGTGCGTTTACCGTGAT
GACTGACAGTCTTCT

*dhbB::*TnJM1 (2)

GAATCAATGTGTTCAGCTTGGGATTCCTGTTGTCTATACCGCACAGCCGGGAAGCCAAAATCCGGATGACCGTGCGCTGCTGAC
AGACTTTTGGGGCCCGGGATTAAACAGCGGTCCTTATGAGGAGAAAATTATAACCGAGCTGGCACCAGAGGATGATGATCTTG
TGCTGACAAAATGGAGATACAGCGCGTTTAAGAGAACGAATCTGCTTGAAATGATGCGCAAAGAGGGACGCGATCAGCTGATC
ATTACAGGAATTTACGCCCATATCGGCTGTCTTGTTACAGCATGTGAAGCATTTATGGAGGATATTAAAGCCTTTTTTGTGGG
AGATGCAGTTGCTGATTTTTCATTAGAAAAACATCAAATGGCGCTGGAATATGCGGCTGGACGCTGTGCGTTTACCGTGATGA
CTGACAGTCTTCTTGATCAGCTGCAGAATGCGCCGGCAGACGTTCAAAAAAACGTCAGCAAACACTGGCAAAAAGAACGTGTTT
AACAGGTTGGCTGATAAGTCCCCGGTCTGGTCGACTAAGTGAGGATTAACTAATAAGGAGGACAAACATGTCAAAAGGAGAA
GAACTTTTTACAGGTGTAGTACCTATCTTGGTTGAATTGGATGGTGATGTTAACGGTCACAAATTTTCTGTACGTGGTGAAGG
TGAAGGTGATGCAACTAACGGTAAATTGACACTTAAATTCATTTGTACAACTGGAAAACTTCCTGTTCCTTGGCCTACTCTTG
TTACAACATTGACATATGGAGTACAATGTTTTTCACGTTATCCTGATCATATGAAACGTCACGATTTTTTTAAATCTGCTATG
CCAGAAGGTTATGTACAAGAACGTACAATTTCATTTAAAGATGACGGAACATATAAAACACGTGCTGAAGTAAAATTCGAAG
GTGACACTCTTGTTAATCGTATCGAATTGAAAGGAATCGATTTCAAAGAAGATGGTAACATTTTGGGACACAAACTTGAATAC
AACTTCAACTCTCATAATGTTTATATCACAGCTGACAAACAAAAAAACGGTATTAAAGCTAATTTTAAAATTCGTCACAATGT
TGAAGATGGATCTGTTCAATTGGCTGATCATTATCAACAAAATACACCAATCGGAGACGGACCAGTATTGCTTCCAGATAACC
ACTACCTTTCTACTCAATCAGTTCTTTCAAAAGATCCTAACGAAAAACGTGACCATATGGTACTTCTTGAATTTGTTACAGCA
GCAGGTATCACTCACGGTATGGACGAACTTTATAAATAAGGATCCTACTCGAGCCACATAGATGGCGTCGCTAGTATTAAATG
CATATTATTTTTATATAGTACCAACCTTCAAATGATTCCCTATAGCTTGTAAATTCTATCATAATTGTGGTTTCAAAATCGGC
TCCGTCGATACTATGTTATACGCCAACTTTGAAAACAACTTTGAAAAAGCTGTTTTCTGGTATTTAAGGTTTTAGAATGCAAG
GAACAGTGAATTGGAGTTCGTCTTGTTATAATTAGCTTCTTGGGGTATCTTTAAATACTGTAGAAAAGAGGAAGGAAATAAT

AAATGGCTAAAATGAGAATATCACCGGAATTGAAAAAACTGATCGAAAAATACCGCTGCGTAAAAGATACGGAAGGAATGTC
TCCTGCTAAGGTATATAAGCTGGTGGGAGAAAATGAAAACCTATATTTAAAAATGACGGACAGCCGGTATAAAGGGACCACCT
ATGATGTAGAACGGGAAAAGGACATGATGCTATGGCTGGAAGGAAAGCTGCCTGTTCCAAAGGTCCTGCACTTTGAACGGCAT
GATGGCTGGAGCAATCTGCTCATGAGTGAGGCCGATGGCGTCCTTTGCTCGGAAGAGTATGAAGATGAACAAAGCCCTGAAAA
GATTATCGAGCTGTATGCGGAGTGCATCAGGCTCTTTCACTCCATCGACATATCGGATTGTCCCTATACGAATAGCTTAGACA
GCCGCTTAGCCGAATTGGATTACTTACTGAATAACGATCTGGCCGATGTGGATTGCGAAAACTGGGAAGAAGACACTCCATTT
AAAGATCCGCGCGAGCTGTATGATTTTTTAAAGACGGAAAAGCCCGAAGAGGAACTTGTCTTTTCCCACGGCGACCTGGGAGA
CAGCAACATCTTTGTGAAAGATGGCAAAGTAAGTGGCTTTATTGATCTTGGGAGAAGCGGCAGGGCGGACAAGTGGTATGACA
TTGCCTTCTGCGTCCGGTCGATCAGGGAGGATATCGGGGAAGAACAGTATGTCGAGCTATTTTTTGACTTACTGGGGATCAAG
CCTGATTGGGAGAAAATAAAATATTATATTTTACTGGATGAATTGTTTTAGTACCTAGATTTAGATGTCTAAAGAATTGATG
CTTTAACTACATGCTTTTTAGACATCTAATCTTTTCTGAAGTACATCCGCAACTGTCCATACTCTGATGTTTTATATCTTTTCT
AAAAGTTCGCTAGATAGGGGTCCCGAGCGCCTACGAGGAATTTGTATCGGGGTATAGATCTAAAACGAATTTTAACAAAAAAA
TGTGTTTTTCTTTGTTCTCGAGCCCGGGGCTAGCACTAGTAGACCGGGGACTTATCAGCCAACCTGTTACATGTGAGAATATCC
GTAAACAAATTGCTGAGCTTCTACAAGAAACACCGGAAGACATCACAGATCAAGAGGATTTGCTCGATCGTGGTCTTGATTCG
GTAAGGATCATGACATTGGTGGAACAATGGCGCCGTGAAGGGGCAGAGGTGACTTTCGTGGAATTGGCTGAACGCCCAACGAT
CGAAGAATGGCAGAAATTGCTCACAACTCGCAGCCAGCAAGTGCTGCCAAACGCGGATTATTTATAAAAGGAGGGTAAACAAA
CGGATGCCTG

*dhbE::*TnJM1

ACGGGAAAAGGTCACGATAACCGCTCTTGTTCCGCCTCTTGCGATGGTATGGATGGATGCGGCATCCTCACGCCGTGATGATTT
ATCCAGCCTTCAAGTGCTGCAGGTCGGCGGTGCCAAGTTTAGTGCTGAAGCCGCGCGCAGGGTAAAAGCTGTTTTCGGCTGCAC
GCTGCAGCAGGTGTTCGGAATGGCAGAGGGTCTCGTCAATTATACGAGATTGGATGATCCTGAGGAGATCATTGTCAACACCC
AAGGAAAACCGATGTCTCCATATGATGAAATGCGTGTTTGGGATGATCATGATCGCGACGTAAAACCTGGTGAAACAGGCCAT
CTGCTGACGCGGGGGCCGTATACAATTCGAGGTTACTAACAGGTTGGCTGATAAGTCCCCGGTCTGGTCGACTAAGTGAGGAT
TAACTAATAAGGAGGACAAACATGTCAAAAGGAGAAGAACTTTTTACAGGTGTAGTACCTATCTTGGTTGAATTGGATGGTG
ATGTTAACGGTCACAAATTTTCTGTACGTGGTGAAGGTGAAGGTGATGCAACTAACGGTAAATTGACACTTAAATTCATTTGT
ACAACTGGAAAACTTCCTGTTCCTTGGCCTACTCTTGTTACAACATTGACATATGGAGTACAATGTTTTTCACGTTATCCTGAT
CATATGAAACGTCACGATTTTTTTAAATCTGCTATGCCAGAAGGTTATGTACAAGAACGTACAATTTCATTTAAAGATGACGG
AACATATAAAACACGTGCTGAAGTAAAATTCGAAGGTGACACTCTTGTTAATCGTATCGAATTGAAAGGAATCGATTTCAAAG
AAGATGGTAACATTTTGGGACACAAACTTGAATACAACTTCAACTCTCATAATGTTTATATCACAGCTGACAAACAAAAAAAC
GGTATTAAAGCTAATTTTAAAATTCGTCACAATGTTGAAGATGGATCTGTTCAATTGGCTGATCATTATCAACAAAATACACC
AATCGGAGACGGACCAGTATTGCTTCCAGATAACCACTACCTTTCTACTCAATCAGTTCTTTCAAAAGATCCTAACGAAAAAC
GTGACCATATGGTACTTCTTGAATTTGTTACAGCAGCAGGTATCACTCACGGTATGGACGAACTTTATAAATAAGGATCCTAC
TCGAGCCACATAGATGGCGTCGCTAGTATTAAATGCATATTATTTTTATATAGTACCAACCTTCAAATGATTCCCTATAGCTT
GTAAATTCTATCATAATTGTGGTTTCAAAATCGGCTCCGTCGATACTATGTTATACGCCAACTTTGAAAACAACTTTGAAAAA
GCTGTTTTCTGGTATTTAAGGTTTTAGAATGCAAGGAACAGTGAATTGGAGTTCGTCTTGTTATAATTAGCTTCTTGGGGTAT
CTTTAAATACTGTAGAAAAGAGGAAGGAAATAATAAATGGCTAAAATGAGAATATCACCGGAATTGAAAAAACTGATCGAAA
AATACCGCTGCGTAAAAGATACGGAAGGAATGTCTCCTGCTAAGGTATATAAGCTGGTGGGAGAAAATGAAAACCTATATTTA
AAAATGACGGACAGCCGGTATAAAGGGACCACCTATGATGTAGAACGGGAAAAGGACATGATGCTATGGCTGGAAGGAAAGC
TGCCTGTTCCAAAGGTCCTGCACTTTGAACGGCATGATGGCTGGAGCAATCTGCTCATGAGTGAGGCCGATGGCGTCCTTTGCT
CGGAAGAGTATGAAGATGAACAAAGCCCTGAAAAGATTATCGAGCTGTATGCGGAGTGCATCAGGCTCTTTCACTCCATCGAC
ATATCGGATTGTCCCTATACGAATAGCTTAGACAGCCGCTTAGCCGAATTGGATTACTTACTGAATAACGATCTGGCCGATGT
GGATTGCGAAAACTGGGAAGAAGACACTCCATTTAAAGATCCGCGCGAGCTGTATGATTTTTTAAAGACGGAAAAGCCCGAAG
AGGAACTTGTCTTTTCCCACGGCGACCTGGGAGACAGCAACATCTTTGTGAAAGATGGCAAAGTAAGTGGCTTTATTGATCTT
GGGAGAAGCGGCAGGGCGGACAAGTGGTATGACATTGCCTTCTGCGTCCGGTCGATCAGGGAGGATATCGGGGAAGAACAGTA
TGTCGAGCTATTTTTTGACTTACTGGGGATCAAGCCTGATTGGGAGAAAATAAAATATTATATTTTACTGGATGAATTGTTTT
AGTACCTAGATTTAGATGTCTAAAGAATTGATGCTTTAACTACATGCTTTTTAGACATCTAATCTTTTCTGAAGTACATCCGC
AACTGTCCATACTCTGATGTTTTATATCTTTTCTAAAAGTTCGCTAGATAGGGGTCCCGAGCGCCTACGAGGAATTTGTATCG
GGGTATAGATCTAAAACGAATTTTAACAAAAAAATGTGTTTTTCTTTGTTCTCGAGCCCGGGGCTAGCACTAGTAGACCGGGG
ACTTATCAGCCAACCTGTTAAGGCAGAAGAGCATAACGCCGCTTCATTTACTGAGGACGGTTTTTACCGTACGGGTGATATCG
TCAGGCTGACACGAGACGGCTATATTGTCGTTGAAGGCCGGGCGAAGGATCAAATTAACCGTGGAGGAGAAAAGGTTGCGGCT
GAAGAGGTAGAAAATCATCTGCTGGCGCATCCGGCTGTCCATGATGCGGCAATGGTCTCCATGCCTGATCAATTTCTTGGCGA
AAGATCTTGTGTGTTCATTATTCCCCGGGATGAAGCCCCAAAAGCCGCAGAGCTTAAAGCATTTTTGAGAGAGCGCGGACTGG
CGGCATATAAAATCCCTGATCGAGTTGAATTTGTCGAATCCTTCCCGCAGACAGGAGTAGGAAAAGTCAGCAAAAAAGCGCTC
CGTGAAGCCATTTCCGAGAAGCTTCTTGCAGGATTTAAAAAATAA

*dhbF::*TnJM1

GCACATCTGTCAGTGATCGGGCCCCGCGGACTCCTCAGGAAGAGATATTGTGTGACTTGTTTGCAGAGGTTCTCGGTTTGGCAC
GCGTCGGTATCGATGACAGTTTCTTCGAGCTTGGGGGCCATTCTCTTCTTGCAGCCCGTCTGATGAGCCGCATTCGCGAGGTAA
TGGGAGCCGAACTTGGTATCGCCAAGCTCTTTGACGAACCGACAGTAGCCGGACTCGCTGCCCATCTTGATTTGGCGCAGAGTG
CATGTCCCGCTTTGCAGAGAGCTGAGCGGCCTGAAAAGATCCCGCTCTCATTTGCCCAGCGCCGGCTATGGTTCCTGCATTGTC
TGGAAGGACCGAGTCCTACTTATAATATTCCGGTTGCTGTCCGTTTGTCAGGTGAGTTGGATCAAGGATTGCTGAAAGCGGCA
CTTTATGACCTCGTCTGCCGTCATGAAAGTCTTCGGACGATCTTCCCTGAATCACAGGGGACATCCTATCAGCATATTTTAACA
GGTTGGCTGATAAGTCCCCGGTCTGGTCGACTAAGTGAGGATTAACTAATAAGGAGGACAAACATGTCAAAAGGAGAAGAACT
TTTTACAGGTGTAGTACCTATCTTGGTTGAATTGGATGGTGATGTTAACGGTCACAAATTTTCTGTACGTGGTGAAGGTGAAG
GTGATGCAACTAACGGTAAATTGACACTTAAATTCATTTGTACAACTGGAAAACTTCCTGTTCCTTGGCCTACTCTTGTTACA
ACATTGACATATGGAGTACAATGTTTTTCACGTTATCCTGATCATATGAAACGTCACGATTTTTTTAAATCTGCTATGCCAGA
AGGTTATGTACAAGAACGTACAATTTCATTTAAAGATGACGGAACATATAAAACACGTGCTGAAGTAAAATTCGAAGGTGAC
ACTCTTGTTAATCGTATCGAATTGAAAGGAATCGATTTCAAAGAAGATGGTAACATTTTGGGACACAAACTTGAATACAACTT
CAACTCTCATAATGTTTATATCACAGCTGACAAACAAAAAAACGGTATTAAAGCTAATTTTAAAATTCGTCACAATGTTGAAG
ATGGATCTGTTCAATTGGCTGATCATTATCAACAAAATACACCAATCGGAGACGGACCAGTATTGCTTCCAGATAACCACTAC
CTTTCTACTCAATCAGTTCTTTCAAAAGATCCTAACGAAAAACGTGACCATATGGTACTTCTTGAATTTGTTACAGCAGCAGG
TATCACTCACGGTATGGACGAACTTTATAAATAAGGATCCTACTCGAGCCACATAGATGGCGTCGCTAGTATTAAATGCATAT
TATTTTTATATAGTACCAACCTTCAAATGATTCCCTATAGCTTGTAAATTCTATCATAATTGTGGTTTCAAAATCGGCTCCGT
CGATACTATGTTATACGCCAACTTTGAAAACAACTTTGAAAAAGCTGTTTTCTGGTATTTAAGGTTTTAGAATGCAAGGAACA
GTGAATTGGAGTTCGTCTTGTTATAATTAGCTTCTTGGGGTATCTTTAAATACTGTAGAAAAGAGGAAGGAAATAATAAATG
GCTAAAATGAGAATATCACCGGAATTGAAAAAACTGATCGAAAAATACCGCTGCGTAAAAGATACGGAAGGAATGTCTCCTGC
TAAGGTATATAAGCTGGTGGGAGAAAATGAAAACCTATATTTAAAAATGACGGACAGCCGGTATAAAGGGACCACCTATGAT
GTAGAACGGGAAAAGGACATGATGCTATGGCTGGAAGGAAAGCTGCCTGTTCCAAAGGTCCTGCACTTTGAACGGCATGATGG
CTGGAGCAATCTGCTCATGAGTGAGGCCGATGGCGTCCTTTGCTCGGAAGAGTATGAAGATGAACAAAGCCCTGAAAAGATTA
TCGAGCTGTATGCGGAGTGCATCAGGCTCTTTCACTCCATCGACATATCGGATTGTCCCTATACGAATAGCTTAGACAGCCGCT
TAGCCGAATTGGATTACTTACTGAATAACGATCTGGCCGATGTGGATTGCGAAAACTGGGAAGAAGACACTCCATTTAAAGAT
CCGCGCGAGCTGTATGATTTTTTAAAGACGGAAAAGCCCGAAGAGGAACTTGTCTTTTCCCACGGCGACCTGGGAGACAGCAA
CATCTTTGTGAAAGATGGCAAAGTAAGTGGCTTTATTGATCTTGGGAGAAGCGGCAGGGCGGACAAGTGGTATGACATTGCCT
TCTGCGTCCGGTCGATCAGGGAGGATATCGGGGAAGAACAGTATGTCGAGCTATTTTTTGACTTACTGGGGATCAAGCCTGAT
TGGGAGAAAATAAAATATTATATTTTACTGGATGAATTGTTTTAGTACCTAGATTTAGATGTCTAAAGAATTGATGCTTTAA
CTACATGCTTTTTAGACATCTAATCTTTTCTGAAGTACATCCGCAACTGTCCATACTCTGATGTTTTATATCTTTTCTAAAAGT
TCGCTAGATAGGGGTCCCGAGCGCCTACGAGGAATTTGTATCGGGGTATAGATCTAAAACGAATTTTAACAAAAAAATGTGTT
TTTCTTTGTTCTCGAGCCCGGGGCTAGCACTAGTAGACCGGGGACTTATCAGCCAACCTGTTAGATGCCGATCGAGCCTGTCCC
GAGTTACATGTTACTGAAATTGCTGAGAAGGAGCTTTCTGATCGGCTTGCCGAAGCTGTACGCTACAGCTTTGATCTTGCAGC
TGAACCTGCTTTTCGTGCCGAGCTTTTTGTGATCGGTCCTGACGAGTACGTGCTGCTTCTCC

*epeE::*TnJM1

ATTTAGATTCAAGGTGTAATGCATCCTGTGACCATTGTTGTTTTTCTAGTTCACCAACTTCAACAACCAGGATGGAAAAAGAA
TACATAAGAGAGTTAGTAACTGAATTTGCAAAAAACAAAACCATTCAAGTGATTTCTTTTACAGGTGGGGAAGTTTTTTTAG
ATTATAAATTTTTAAAAGAACTAATGGAAATCATTAAACCATATGAAAAACAAATAACGTTGATCTCTAATGGATTTTGGGG
TTTGAGCAAGAAAAAGGTCCAGGAGTACTTTCATGATATGAATTCTTTAAATGTGATAGCACTGACTATAAGTTATGATGAGT
ACCATGCTCCATTTGTCAAGTCTTCCAGTATTAAAAACATATTAGAACATAGCAGGAAATACCCTGATATCGATATTTCTCTT
AATATGGCAGTTACCAAAGACAAAATGTCCAACCACATTTTAGAGGAATTGGGGGATTCAATCTTAGGTGTAAAGATAACAA
AATTTCCAATGATATCAGTTGGCGCTGCTAAAACTAGAATTAAGCAAGAAAACATACATAAATTTTACAGTCTTGAAGATGAA
GATTCCTTACATTGTCCCGGATATGACATTGTAACAGGTTGGCTGATAAGTCCCCGGTCTGGTCGACTAAGTGAGGATTAACT
AATAAGGAGGACAAACATGTCAAAAGGAGAAGAACTTTTTACAGGTGTAGTACCTATCTTGGTTGAATTGGATGGTGATGTT
AACGGTCACAAATTTTCTGTACGTGGTGAAGGTGAAGGTGATGCAACTAACGGTAAATTGACACTTAAATTCATTTGTACAAC
TGGAAAACTTCCTGTTCCTTGGCCTACTCTTGTTACAACATTGACATATGGAGTACAATGTTTTTCACGTTATCCTGATCATAT
GAAACGTCACGATTTTTTTAAATCTGCTATGCCAGAAGGTTATGTACAAGAACGTACAATTTCATTTAAAGATGACGGAACAT
ATAAAACACGTGCTGAAGTAAAATTCGAAGGTGACACTCTTGTTAATCGTATCGAATTGAAAGGAATCGATTTCAAAGAAGA
TGGTAACATTTTGGGACACAAACTTGAATACAACTTCAACTCTCATAATGTTTATATCACAGCTGACAAACAAAAAAACGGTA
TTAAAGCTAATTTTAAAATTCGTCACAATGTTGAAGATGGATCTGTTCAATTGGCTGATCATTATCAACAAAATACACCAATC
GGAGACGGACCAGTATTGCTTCCAGATAACCACTACCTTTCTACTCAATCAGTTCTTTCAAAAGATCCTAACGAAAAACGTGA
CCATATGGTACTTCTTGAATTTGTTACAGCAGCAGGTATCACTCACGGTATGGACGAACTTTATAAATAAGGATCCTACTCGA
GCCACATAGATGGCGTCGCTAGTATTAAATGCATATTATTTTTATATAGTACCAACCTTCAAATGATTCCCTATAGCTTGTAA
ATTCTATCATAATTGTGGTTTCAAAATCGGCTCCGTCGATACTATGTTATACGCCAACTTTGAAAACAACTTTGAAAAAGCTG
TTTTCTGGTATTTAAGGTTTTAGAATGCAAGGAACAGTGAATTGGAGTTCGTCTTGTTATAATTAGCTTCTTGGGGTATCTTT
AAATACTGTAGAAAAGAGGAAGGAAATAATAAATGGCTAAAATGAGAATATCACCGGAATTGAAAAAACTGATCGAAAAATA
CCGCTGCGTAAAAGATACGGAAGGAATGTCTCCTGCTAAGGTATATAAGCTGGTGGGAGAAAATGAAAACCTATATTTAAAA
ATGACGGACAGCCGGTATAAAGGGACCACCTATGATGTAGAACGGGAAAAGGACATGATGCTATGGCTGGAAGGAAAGCTGCC

TGTTCCAAAGGTCCTGCACTTTGAACGGCATGATGGCTGGAGCAATCTGCTCATGAGTGAGGCCGATGGCGTCCTTTGCTCGGA
AGAGTATGAAGATGAACAAAGCCCTGAAAAGATTATCGAGCTGTATGCGGAGTGCATCAGGCTCTTTCACTCCATCGACATAT
CGGATTGTCCCTATACGAATAGCTTAGACAGCCGCTTAGCCGAATTGGATTACTTACTGAATAACGATCTGGCCGATGTGGAT
TGCGAAAACTGGGAAGAAGACACTCCATTTAAAGATCCGCGCGAGCTGTATGATTTTTTAAAGACGGAAAAGCCCGAAGAGGA
ACTTGTCTTTTCCCACGGCGACCTGGGAGACAGCAACATCTTTGTGAAAGATGGCAAAGTAAGTGGCTTTATTGATCTTGGGA
GAAGCGGCAGGGCGGACAAGTGGTATGACATTGCCTTCTGCGTCCGGTCGATCAGGGAGGATATCGGGGAAGAACAGTATGTC
GAGCTATTTTTTGACTTACTGGGGATCAAGCCTGATTGGGAGAAAATAAAATATTATATTTTACTGGATGAATTGTTTTAGTA
CCTAGATTTAGATGTCTAAAGAATTGATGCTTTAACTACATGCTTTTTAGACATCTAATCTTTTCTGAAGTACATCCGCAACT
GTCCATACTCTGATGTTTTATATCTTTTCTAAAAGTTCGCTAGATAGGGGTCCCGAGCGCCTACGAGGAATTTGTATCGGGGT
ATAGATCTAAAACGAATTTTAACAAAAAAATGTGTTTTTCTTTGTTCTCGAGCCCGGGGCTAGCACTAGTAGACCGGGGACTT
ATCAGCCAACCTGTTATATCATCATGATGGAGAAATTTATCCATGTTGTTCCCCTGCTATTTTTGAAACGAAAATAACCCTAA
GGGAAGAATATAACCAAAGTTTCGAAAGAACCGTGGAAAAGTTAAATTCCAATTTGCTATTATTTATTCTTAGAAAAGAAGG
ATTCAAATGGTTTTTGAATATTTTAAAAGAAAATAACAAAATAGAGGAATTTGACATTCCTTATGAATTTTCCTCTATTTGTG
GAGTTTGTGGTTCCTTATTCAACTCTGCAGAAAAAATAAACTACTTCTATCCTTATATGGAAAAATACTATAATGAAAATTTT
AAAGTATGAAGATGAAAAATATGAAGTGCTTGTGCAAAACAACG

*flgK::*TnJM1

GGGTCTCATTGGAGGCAACTGACTATTTCCCTGCTGTATCTAAAAATGCAGAAAAAACAGCGGGACAAATGGGTACGGGCGTT
CAAGGAAAATCAGTTGAGAGAATAAGAGATATCTTTCTTGACTACCAATACCGTCTTCAAAACAACAGTGCCGGATACTATGA
CACGAAGGCAAAAGCGCTGTCCCAAATGGAAGGCGTTTTAAATGAAACGGATGACAGCGGCTTGAACAGTGTGCTCAATTCGT
TTTGGAATTCCCTGCAGGAATTATCGAATAATACAAATGAAGAAAGTGCACGTTCTGTTGTTGCTCGAAAAGGACAAGCTGTA
GCTGAAACGTTTAATTATATTTCTGAATCACTTACAAATGTCCAATCGAATTTAAAAGCAGAGCTAAATACAACTGTGTTGGA
TGTCAATTCTCTGCTTTCTCAGTTAAACAGTTTAAATAAGCAAATTGCACAAGTAGAGCCGGTCGGGCTTCTTCCGAATGGCT
TAACAGGTTGGCTGATAAGTCCCCGGTCTGGTCGACTAAGTGAGGATTAACTAATAAGGAGGACAAACATGTCAAAAGGAGAA
GAACTTTTTACAGGTGTAGTACCTATCTTGGTTGAATTGGATGGTGATGTTAACGGTCACAAATTTTCTGTACGTGGTGAAGG
TGAAGGTGATGCAACTAACGGTAAATTGACACTTAAATTCATTTGTACAACTGGAAAACTTCCTGTTCCTTGGCCTACTCTTG
TTACAACATTGACATATGGAGTACAATGTTTTTCACGTTATCCTGATCATATGAAACGTCACGATTTTTTTAAATCTGCTATG
CCAGAAGGTTATGTACAAGAACGTACAATTTCATTTAAAGATGACGGAACATATAAAACACGTGCTGAAGTAAAATTCGAAG
GTGACACTCTTGTTAATCGTATCGAATTGAAAGGAATCGATTTCAAAGAAGATGGTAACATTTTGGGACACAAACTTGAATAC
AACTTCAACTCTCATAATGTTTATATCACAGCTGACAAACAAAAAAACGGTATTAAAGCTAATTTTAAAATTCGTCACAATGT
TGAAGATGGATCTGTTCAATTGGCTGATCATTATCAACAAAATACACCAATCGGAGACGGACCAGTATTGCTTCCAGATAACC
ACTACCTTTCTACTCAATCAGTTCTTTCAAAAGATCCTAACGAAAAACGTGACCATATGGTACTTCTTGAATTTGTTACAGCA
GCAGGTATCACTCACGGTATGGACGAACTTTATAAATAAGGATCCTACTCGAGCCACATAGATGGCGTCGCTAGTATTAAATG
CATATTATTTTTATATAGTACCAACCTTCAAATGATTCCCTATAGCTTGTAAATTCTATCATAATTGTGGTTTCAAAATCGGC
TCCGTCGATACTATGTTATACGCCAACTTTGAAAACAACTTTGAAAAAGCTGTTTTCTGGTATTTAAGGTTTTAGAATGCAAG
GAACAGTGAATTGGAGTTCGTCTTGTTATAATTAGCTTCTTGGGGTATCTTTAAATACTGTAGAAAAGAGGAAGGAAATAAT
AAATGGCTAAAATGAGAATATCACCGGAATTGAAAAAACTGATCGAAAAATACCGCTGCGTAAAAGATACGGAAGGAATGTC
TCCTGCTAAGGTATATAAGCTGGTGGGAGAAAATGAAAACCTATATTTAAAAATGACGGACAGCCGGTATAAAGGGACCACCT
ATGATGTAGAACGGGAAAAGGACATGATGCTATGGCTGGAAGGAAAGCTGCCTGTTCCAAAGGTCCTGCACTTTGAACGGCAT
GATGGCTGGAGCAATCTGCTCATGAGTGAGGCCGATGGCGTCCTTTGCTCGGAAGAGTATGAAGATGAACAAAGCCCTGAAAA
GATTATCGAGCTGTATGCGGAGTGCATCAGGCTCTTTCACTCCATCGACATATCGGATTGTCCCTATACGAATAGCTTAGACA
GCCGCTTAGCCGAATTGGATTACTTACTGAATAACGATCTGGCCGATGTGGATTGCGAAAACTGGGAAGAAGACACTCCATTT
AAAGATCCGCGCGAGCTGTATGATTTTTTAAAGACGGAAAAGCCCGAAGAGGAACTTGTCTTTTCCCACGGCGACCTGGGAGA
CAGCAACATCTTTGTGAAAGATGGCAAAGTAAGTGGCTTTATTGATCTTGGGAGAAGCGGCAGGGCGGACAAGTGGTATGACA
TTGCCTTCTGCGTCCGGTCGATCAGGGAGGATATCGGGGAAGAACAGTATGTCGAGCTATTTTTTGACTTACTGGGGATCAAG
CCTGATTGGGAGAAAATAAAATATTATATTTTACTGGATGAATTGTTTTAGTACCTAGATTTAGATGTCTAAAGAATTGATG
CTTTAACTACATGCTTTTTAGACATCTAATCTTTTCTGAAGTACATCCGCAACTGTCCATACTCTGATGTTTTATATCTTTTCT
AAAAGTTCGCTAGATAGGGGTCCCGAGCGCCTACGAGGAATTTGTATCGGGGTATAGATCTAAAACGAATTTTAACAAAAAAA
TGTGTTTTTCTTTGTTCTCGAGCCCGGGGCTAGCACTAGTAGACCGGGGACTTATCAGCCAACCTGTTATATGACCAGCGAGAT
TTGCTGATCGATAAATTATCCTCAATGGTTGATATCAAAGTCAGCTACAACAAATCAGGCGGGAATGCACTTGCATCTGCTGA
AGGAACTGTATCAATTGAAATCCTTGATAAAAATAAACAGTCGCTAGGAACTGTTTTGGACGGTAAAAATTACGAGGTTTCAG
AACTTGCAGCCAACTATGATAATGAAACTGGGTTAGTATCAAGTATTTCAATCGGGGATACAGCTGTACAGGCGGAGTCCTTT
AGCAGCAAGGGATCCCTTTTAGGGTTTATAGAGTCTTATGGGTATATAACAGCAGATGGCCAAGAAAAGGGCGTATACCCGGA
AATGCTTTCTGATCTTGACAATATGGCGCTTGAATTCGCGAAAGCTTTCAATGAAGTGCACAGAAATGGTGTGACCAAGAGCG
GTGAACAAGGCGGAGACTTTTTTGATTTTACTGGCGGTGAAACTGAACCTGCCAAGGGCGCGGCGGGC

*hag::*TnJM1 (1)

GGATCTCCGCATTATCCTCACAAAAAAAGTGAGGATTTTTTTATTTTTGTATTAACAAAATCAGAGACAATCCGATATTAATG
ATGTAGCCGGGAGGAGGCGCAAAAGACTCAGCCAGTTACAAAATAAGGGCACAAGGACGTGCCTTAACAACATATTCAGGGAG
GAACAAAACAATGAGAATTAACCACAATATTGCAGCGCTTAACACACTGAACCGTTTGTCTTCAAACAACAGTGCGAGCCAAA
AGAACATGGAGAAACTTTCTTCAGGTCTTCGCATCAACCGTGCGGGAGATGACGCAGCAGGTCTTGCGATCTCTGAAAAAATG
AGAGGACAAATCAGAGGTCTTGAAATGGCTTCTAAAAACTCTCAAGACGGAATCTCTCTTATCCAAACAGCTGAGGGTGCATT
AACTGAAACTCATGCGATCCTTCAACGTGTTCGTGAGCTAGTTGTTCAAGCTGGAAACACTGGAACTCAGGACAAAGCAACTG
ATTTGCAATCTAACAGGTTGGCTGATAAGTCCCCGGTCTGGTCGACTAAGTGAGGATTAACTAATAAGGAGGACAAACATGTC
AAAAGGAGAAGAACTTTTTACAGGTGTAGTACCTATCTTGGTTGAATTGGATGGTGATGTTAACGGTCACAAATTTTCTGTAC
GTGGTGAAGGTGAAGGTGATGCAACTAACGGTAAATTGACACTTAAATTCATTTGTACAACTGGAAAACTTCCTGTTCCTTGG
CCTACTCTTGTTACAACATTGACATATGGAGTACAATGTTTTTCACGTTATCCTGATCATATGAAACGTCACGATTTTTTTAA
ATCTGCTATGCCAGAAGGTTATGTACAAGAACGTACAATTTCATTTAAAGATGACGGAACATATAAAACACGTGCTGAAGTAA
AATTCGAAGGTGACACTCTTGTTAATCGTATCGAATTGAAAGGAATCGATTTCAAAGAAGATGGTAACATTTTGGGACACAAA
CTTGAATACAACTTCAACTCTCATAATGTTTATATCACAGCTGACAAACAAAAAAACGGTATTAAAGCTAATTTTAAAATTCG
TCACAATGTTGAAGATGGATCTGTTCAATTGGCTGATCATTATCAACAAAATACACCAATCGGAGACGGACCAGTATTGCTTC
CAGATAACCACTACCTTTCTACTCAATCAGTTCTTTCAAAAGATCCTAACGAAAAACGTGACCATATGGTACTTCTTGAATTT
GTTACAGCAGCAGGTATCACTCACGGTATGGACGAACTTTATAAATAAGGATCCTACTCGAGCCACATAGATGGCGTCGCTAG
TATTAAATGCATATTATTTTTATATAGTACCAACCTTCAAATGATTCCCTATAGCTTGTAAATTCTATCATAATTGTGGTTTC
AAAATCGGCTCCGTCGATACTATGTTATACGCCAACTTTGAAAACAACTTTGAAAAAGCTGTTTTCTGGTATTTAAGGTTTTA
GAATGCAAGGAACAGTGAATTGGAGTTCGTCTTGTTATAATTAGCTTCTTGGGGTATCTTTAAATACTGTAGAAAAGAGGAA
GGAAATAATAAATGGCTAAAATGAGAATATCACCGGAATTGAAAAAACTGATCGAAAAATACCGCTGCGTAAAAGATACGGA
AGGAATGTCTCCTGCTAAGGTATATAAGCTGGTGGGAGAAAATGAAAACCTATATTTAAAAATGACGGACAGCCGGTATAAA
GGGACCACCTATGATGTAGAACGGGAAAAGGACATGATGCTATGGCTGGAAGGAAAGCTGCCTGTTCCAAAGGTCCTGCACTT
TGAACGGCATGATGGCTGGAGCAATCTGCTCATGAGTGAGGCCGATGGCGTCCTTTGCTCGGAAGAGTATGAAGATGAACAAA
GCCCTGAAAAGATTATCGAGCTGTATGCGGAGTGCATCAGGCTCTTTCACTCCATCGACATATCGGATTGTCCCTATACGAAT
AGCTTAGACAGCCGCTTAGCCGAATTGGATTACTTACTGAATAACGATCTGGCCGATGTGGATTGCGAAAACTGGGAAGAAGA
CACTCCATTTAAAGATCCGCGCGAGCTGTATGATTTTTTAAAGACGGAAAAGCCCGAAGAGGAACTTGTCTTTTCCCACGGCG
ACCTGGGAGACAGCAACATCTTTGTGAAAGATGGCAAAGTAAGTGGCTTTATTGATCTTGGGAGAAGCGGCAGGGCGGACAAG
TGGTATGACATTGCCTTCTGCGTCCGGTCGATCAGGGAGGATATCGGGGAAGAACAGTATGTCGAGCTATTTTTTGACTTACT
GGGGATCAAGCCTGATTGGGAGAAAATAAAATATTATATTTTACTGGATGAATTGTTTTAGTACCTAGATTTAGATGTCTAA
AGAATTGATGCTTTAACTACATGCTTTTTAGACATCTAATCTTTTCTGAAGTACATCCGCAACTGTCCATACTCTGATGTTTT
ATATCTTTTCTAAAAGTTCGCTAGATAGGGGTCCCGAGCGCCTACGAGGAATTTGTATCGGGGTATAGATCTAAAACGAATTT
TAACAAAAAAATGTGTTTTTCTTTGTTCTCGAGCCCGGGGCTAGCACTAGTAGACCGGGGACTTATCAGCCAACCTGTTATTC
AAGATGAAATTTCAGCTTTAACAGATGAAATCGATGGTATTTCAAATCGTACAGAATTCAATGGTAAGAAATTGCTCGATGGC
ACTTACAAAGTTGACACAGCTACTCCTGCAAATCAAAAGAACTTGGTATTCCAAATCGGAGCAAATGCTACACAGCAAATCTC
TGTAAATATTGAGGATATGGGTGCTGACGCTCTTGGAATTAAAGAAGCTGATGGTTCAATTGCAGCTCTTCATTCAGTTAATG
ATCTTGACGTAACAAAATTCGCAGATAATGCAGCAGATACTGCTGATATCGGTTTCGATGCTCAATTGAAAGTTGTTGATGAA
GCGATCAACCAAGTTTCTTCTCAACGTGCTAAGCTTGGTGCGGTACAAAATCGTCTAGAGCACACAATTAACAACTTAAGCGC
TTCTGGTGAAAACTTGACAGCTGCTGAGTCTCGTATCCGTGACGTTGACATGGCTAAAGAGATGAGCGAATTCACAAAGA

*hag::*TnJM1 (2)

GTGATCTCCGCATTATCCTCACAAAAAAAGTGAGGATTTTTTTATTTTTGTATTAACAAAATCAGAGACAATCCGATATTAAT
GATGTAGCCGGGAGGAGGCGCAAAAGACTCAGCCAGTTACAAAATAAGGGCACAAGGACGTGCCTTAACAACATATTCAGGGA
GGAACAAAACAATGAGAATTAACCACAATATTGCAGCGCTTAACACACTGAACCGTTTGTCTTCAAACAACAGTGCGAGCCAA
AAGAACATGGAGAAACTTTCTTCAGGTCTTCGCATCAACCGTGCGGGAGATGACGCAGCAGGTCTTGCGATCTCTGAAAAAAT
GAGAGGACAAATCAGAGGTCTTGAAATGGCTTCTAAAAACTCTCAAGACGGAATCTCTCTTAACAGGTTGGCTGATAAGTCCC
CGGTCTGGTCGACTAAGTGAGGATTAACTAATAAGGAGGACAAACATGTCAAAAGGAGAAGAACTTTTTACAGGTGTAGTACC
TATCTTGGTTGAATTGGATGGTGATGTTAACGGTCACAAATTTTCTGTACGTGGTGAAGGTGAAGGTGATGCAACTAACGGTA
AATTGACACTTAAATTCATTTGTACAACTGGAAAACTTCCTGTTCCTTGGCCTACTCTTGTTACAACATTGACATATGGAGTA
CAATGTTTTTCACGTTATCCTGATCATATGAAACGTCACGATTTTTTTAAATCTGCTATGCCAGAAGGTTATGTACAAGAACG
TACAATTTCATTTAAAGATGACGGAACATATAAAACACGTGCTGAAGTAAAATTCGAAGGTGACACTCTTGTTAATCGTATCG
AATTGAAAGGAATCGATTTCAAAGAAGATGGTAACATTTTGGGACACAAACTTGAATACAACTTCAACTCTCATAATGTTTAT
ATCACAGCTGACAAACAAAAAAACGGTATTAAAGCTAATTTTAAAATTCGTCACAATGTTGAAGATGGATCTGTTCAATTGGC
TGATCATTATCAACAAAATACACCAATCGGAGACGGACCAGTATTGCTTCCAGATAACCACTACCTTTCTACTCAATCAGTTCT
TTCAAAAGATCCTAACGAAAAACGTGACCATATGGTACTTCTTGAATTTGTTACAGCAGCAGGTATCACTCACGGTATGGACG
AACTTTATAAATAAGGATCCTACTCGAGCCACATAGATGGCGTCGCTAGTATTAAATGCATATTATTTTTATATAGTACCAAC
CTTCAAATGATTCCCTATAGCTTGTAAATTCTATCATAATTGTGGTTTCAAAATCGGCTCCGTCGATACTATGTTATACGCCA
ACTTTGAAAACAACTTTGAAAAAGCTGTTTTCTGGTATTTAAGGTTTTAGAATGCAAGGAACAGTGAATTGGAGTTCGTCTTG
TTATAATTAGCTTCTTGGGGTATCTTTAAATACTGTAGAAAAGAGGAAGGAAATAATAAATGGCTAAAATGAGAATATCACC
GGAATTGAAAAAACTGATCGAAAAATACCGCTGCGTAAAAGATACGGAAGGAATGTCTCCTGCTAAGGTATATAAGCTGGTG

GGAGAAAATGAAAACCTATATTTAAAAATGACGGACAGCCGGTATAAAGGGACCACCTATGATGTAGAACGGGAAAAGGACA
TGATGCTATGGCTGGAAGGAAAGCTGCCTGTTCCAAAGGTCCTGCACTTTGAACGGCATGATGGCTGGAGCAATCTGCTCATG
AGTGAGGCCGATGGCGTCCTTTGCTCGGAAGAGTATGAAGATGAACAAAGCCCTGAAAAGATTATCGAGCTGTATGCGGAGTG
CATCAGGCTCTTTCACTCCATCGACATATCGGATTGTCCCTATACGAATAGCTTAGACAGCCGCTTAGCCGAATTGGATTACTT
ACTGAATAACGATCTGGCCGATGTGGATTGCGAAAACTGGGAAGAAGACACTCCATTTAAAGATCCGCGCGAGCTGTATGATT
TTTTAAAGACGGAAAAGCCCGAAGAGGAACTTGTCTTTTCCCACGGCGACCTGGGAGACAGCAACATCTTTGTGAAAGATGGC
AAAGTAAGTGGCTTTATTGATCTTGGGAGAAGCGGCAGGGCGGACAAGTGGTATGACATTGCCTTCTGCGTCCGGTCGATCAG
GGAGGATATCGGGGAAGAACAGTATGTCGAGCTATTTTTTGACTTACTGGGGATCAAGCCTGATTGGGAGAAAATAAAATAT
TATATTTTACTGGATGAATTGTTTTAGTACCTAGATTTAGATGTCTAAAGAATTGATGCTTTAACTACATGCTTTTTAGACAT
CTAATCTTTTCTGAAGTACATCCGCAACTGTCCATACTCTGATGTTTTATATCTTTTCTAAAAGTTCGCTAGATAGGGGTCCCG
AGCGCCTACGAGGAATTTGTATCGGGGTATAGATCTAAAACGAATTTTAACAAAAAAATGTGTTTTTCTTTGTTCTCGAGCCC
GGGGCTAGCACTAGTAGACCGGGGACTTATCAGCCAACCTGTTATCCAAACAGCTGAGGGTGCATTAACTGAAACTCATGCGA
TCCTTCAACGTGTTCGTGAGCTAGTTGTTCAAGCTGGAAACACTGGAACTCAGGACAAAGCAACTGATTTGCAATCTATTCAA
GATGAAATTTCAGCTTTAACAGATGAAATCGATGGTATTTCAAATCGTACAGAATTCAATGGTAAGAAATTGCTCGATGGCAC
TTACAAAGTTGACACAGCTACTCCTGCAAATCAAAAGAACTTGGTATTCCAAATCGGAGCAAATGCTACACAGCAAATCTCTG
TAAATATTGAGGATATGGGTGCTGACGCTCTTGGAATTAAAGAAGCTGATGGTTCAATTGCAGCTCTTCATTCAGTTAATGAT
CTTGACGTAACAAAATTCGCAGATAATGCAGCAGATACTGCTGATATCGGTTTCGATGCTCAATTGAAAGTTGTTGATGAAGC
GATCAACCAAGTTTCTTCTCAACGTGCTAAGCTTGGTGCGGTAC

*lytA::*TnJM1

GCGTTCATCCACACCCATGATTAAGACGGAAAAAGGATCTTTTTTATTGATACTGACCTCTTTATCCCTCTTTTTAGATTTATC
AATGCTTTCATGAATACTTGCCACGGTTGAAGCTGCTTTATGCCATAAGTAGTATGCATAGCCGCCAGTTCCGAGTACAAGAA
GTCCGATTATCGTTAAAATTGTCAGTAATAAAGTTTTTTTCTTTTTTCTGCGTTCGTTTCTCATCCTTTGCACCTCGTCTGTTA
AATTACTTTCATTATGAGTTAAATTTCCAATAAATACAACTCTATTTAATACAAGAATGAAATCCTAAAATAGTCGTTTTTTA
TAAAAAAAAGTTTCATTGTTTCTTAATTTTTCTTTAAAATATAAAATAGGTTGACGATAAAATATAATGAGGTGAAAAAAAT
GAAAAAATTTATTGCTTTACTGTTCTTTATATTGCTTCTTTCGGGTTGCGGGGTTAATAGTCAAAAGAGTCAAGGTGAAGATG
TATCGCCAGACAGTAACAGGTTGGCTGATAAGTCCCCGGTCTGGTCGACTAAGTGAGGATTAACTAATAAGGAGGACAAACAT
GTCAAAAGGAGAAGAACTTTTTACAGGTGTAGTACCTATCTTGGTTGAATTGGATGGTGATGTTAACGGTCACAAATTTTCTG
TACGTGGTGAAGGTGAAGGTGATGCAACTAACGGTAAATTGACACTTAAATTCATTTGTACAACTGGAAAACTTCCTGTTCCT
TGGCCTACTCTTGTTACAACATTGACATATGGAGTACAATGTTTTTCACGTTATCCTGATCATATGAAACGTCACGATTTTTT
TAAATCTGCTATGCCAGAAGGTTATGTACAAGAACGTACAATTTCATTTAAAGATGACGGAACATATAAAACACGTGCTGAAG
TAAAATTCGAAGGTGACACTCTTGTTAATCGTATCGAATTGAAAGGAATCGATTTCAAAGAAGATGGTAACATTTTGGGACAC
AAACTTGAATACAACTTCAACTCTCATAATGTTTATATCACAGCTGACAAACAAAAAAACGGTATTAAAGCTAATTTTAAAAT
TCGTCACAATGTTGAAGATGGATCTGTTCAATTGGCTGATCATTATCAACAAAATACACCAATCGGAGACGGACCAGTATTGC
TTCCAGATAACCACTACCTTTCTACTCAATCAGTTCTTTCAAAAGATCCTAACGAAAAACGTGACCATATGGTACTTCTTGAA
TTTGTTACAGCAGCAGGTATCACTCACGGTATGGACGAACTTTATAAATAAGGATCCTACTCGAGCCACATAGATGGCGTCGC
TAGTATTAAATGCATATTATTTTTATATAGTACCAACCTTCAAATGATTCCCTATAGCTTGTAAATTCTATCATAATTGTGGT
TTCAAAATCGGCTCCGTCGATACTATGTTATACGCCAACTTTGAAAACAACTTTGAAAAAGCTGTTTTCTGGTATTTAAGGTT
TTAGAATGCAAGGAACAGTGAATTGGAGTTCGTCTTGTTATAATTAGCTTCTTGGGGTATCTTTAAATACTGTAGAAAAGAGG
AAGGAAATAATAAATGGCTAAAATGAGAATATCACCGGAATTGAAAAAACTGATCGAAAAATACCGCTGCGTAAAAGATACG
GAAGGAATGTCTCCTGCTAAGGTATATAAGCTGGTGGGAGAAAATGAAAACCTATATTTAAAAATGACGGACAGCCGGTATA
AAGGGACCACCTATGATGTAGAACGGGAAAAGGACATGATGCTATGGCTGGAAGGAAAGCTGCCTGTTCCAAAGGTCCTGCAC
TTTGAACGGCATGATGGCTGGAGCAATCTGCTCATGAGTGAGGCCGATGGCGTCCTTTGCTCGGAAGAGTATGAAGATGAACA
AAGCCCTGAAAAGATTATCGAGCTGTATGCGGAGTGCATCAGGCTCTTTCACTCCATCGACATATCGGATTGTCCCTATACGA
ATAGCTTAGACAGCCGCTTAGCCGAATTGGATTACTTACTGAATAACGATCTGGCCGATGTGGATTGCGAAAACTGGGAAGAA
GACACTCCATTTAAAGATCCGCGCGAGCTGTATGATTTTTTAAAGACGGAAAAGCCCGAAGAGGAACTTGTCTTTTCCCACGG
CGACCTGGGAGACAGCAACATCTTTGTGAAAGATGGCAAAGTAAGTGGCTTTATTGATCTTGGGAGAAGCGGCAGGGCGGACA
AGTGGTATGACATTGCCTTCTGCGTCCGGTCGATCAGGGAGGATATCGGGGAAGAACAGTATGTCGAGCTATTTTTTGACTTA
CTGGGGATCAAGCCTGATTGGGAGAAAATAAAATATTATATTTTACTGGATGAATTGTTTTAGTACCTAGATTTAGATGTCTA
AAGAATTGATGCTTTAACTACATGCTTTTTAGACATCTAATCTTTTCTGAAGTACATCCGCAACTGTCCATACTCTGATGTTT
TATATCTTTTCTAAAAGTTCGCTAGATAGGGGTCCCGAGCGCCTACGAGGAATTTGTATCGGGGTATAGATCTAAAACGAATT
TTAACAAAAAAATGTGTTTTTCTTTGTTCTCGAGCCCGGGGCTAGCACTAGTAGACCGGGGACTTATCAGCCAACCTGTTAAC
ATTGAAACAAAAGAAGGTACTTATGTAGGGTTAGCTGATACTCATACAATAGAAGTAACAGTAGATAATGAGCCGGTTAGTC
TTGATATCACTGAAGAATCGACAAGTGATCTTGATAAGTTTAACAGTGGAGATAAGGTCACGATTACATATGAAAAAAATGA
TGAGGGTCAGCTTCTGTTAAAAGATATTGAACGTGCCAACTAAGATATAGGGAGGAACCTGAAATTTTGAAATCTTGCAAACA
ATTGATAGTGTGTTCACTTGCTGCAATCTTATTATTGATTCCATCAGTTTCTTTTGCAGCAGACTCAAACATCTCAGTTAAAT
TG

*lytF::*TnJM1

CTGTTTACACAGTGAAAAGCGGAGATTCACTTTGGCTGATCGCAAATGAGTTTAAAATGACGGTGCAGGAACTGAAAAAATTA
AACGGACTAAGCAGCGATTTAATTCGTGCTGGGCAGAAATTAAAGGTGTCCGGTACTGTTTCTTCAAGCTCTAGTTCCAGTAA
AAAAAGCAACTCTAATAAGAGTTCAAGCTCCAGTTCAAAAAGCAGTTCTAATAAGAGTTCAAGTTCTTCATCTTCTACGGGAA
CATATAAGGTGCAGCTTGGAGATTCACTTTGGAAAATTGCAAACAAAGTCAATATGTCCATCGCTGAATTGAAGGTCTTGAAC
AACTTAAAATCAGACACCATTTACGTGAATCAGGTGTTGAAAACAAAATCAAGCGGCTCTGATACGTCTTCTAAAGACAATTC
ATCCAAATCGAACCAAACTTCAGCAACAACTAAATATACCGTTAAAAGCGGTGATTCACTTTGGAAAATCGCAAACAACTAAC
AGGTTGGCTGATAAGTCCCCGGTCTGGTCGACTAAGTGAGGATTAACTAATAAGGAGGACAAACATGTCAAAAGGAGAAGAA
CTTTTTACAGGTGTAGTACCTATCTTGGTTGAATTGGATGGTGATGTTAACGGTCACAAATTTTCTGTACGTGGTGAAGGTGA
AGGTGATGCAACTAACGGTAAATTGACACTTAAATTCATTTGTACAACTGGAAAACTTCCTGTTCCTTGGCCTACTCTTGTTA
CAACATTGACATATGGAGTACAATGTTTTTCACGTTATCCTGATCATATGAAACGTCACGATTTTTTTAAATCTGCTATGCCA
GAAGGTTATGTACAAGAACGTACAATTTCATTTAAAGATGACGGAACATATAAAACACGTGCTGAAGTAAAATTCGAAGGTG
ACACTCTTGTTAATCGTATCGAATTGAAAGGAATCGATTTCAAAGAAGATGGTAACATTTTGGGACACAAACTTGAATACAAC
TTCAACTCTCATAATGTTTATATCACAGCTGACAAACAAAAAAACGGTATTAAAGCTAATTTTAAAATTCGTCACAATGTTGA
AGATGGATCTGTTCAATTGGCTGATCATTATCAACAAAATACACCAATCGGAGACGGACCAGTATTGCTTCCAGATAACCACT
ACCTTTCTACTCAATCAGTTCTTTCAAAAGATCCTAACGAAAAACGTGACCATATGGTACTTCTTGAATTTGTTACAGCAGCA
GGTATCACTCACGGTATGGACGAACTTTATAAATAAGGATCCTACTCGAGCCACATAGATGGCGTCGCTAGTATTAAATGCAT
ATTATTTTTATATAGTACCAACCTTCAAATGATTCCCTATAGCTTGTAAATTCTATCATAATTGTGGTTTCAAAATCGGCTCC
GTCGATACTATGTTATACGCCAACTTTGAAAACAACTTTGAAAAAGCTGTTTTCTGGTATTTAAGGTTTTAGAATGCAAGGAA
CAGTGAATTGGAGTTCGTCTTGTTATAATTAGCTTCTTGGGGTATCTTTAAATACTGTAGAAAAGAGGAAGGAAATAATAAA
TGGCTAAAATGAGAATATCACCGGAATTGAAAAAACTGATCGAAAAATACCGCTGCGTAAAAGATACGGAAGGAATGTCTCCT
GCTAAGGTATATAAGCTGGTGGGAGAAAATGAAAACCTATATTTAAAAATGACGGACAGCCGGTATAAAGGGACCACCTATG
ATGTAGAACGGGAAAAGGACATGATGCTATGGCTGGAAGGAAAGCTGCCTGTTCCAAAGGTCCTGCACTTTGAACGGCATGAT
GGCTGGAGCAATCTGCTCATGAGTGAGGCCGATGGCGTCCTTTGCTCGGAAGAGTATGAAGATGAACAAAGCCCTGAAAAGAT
TATCGAGCTGTATGCGGAGTGCATCAGGCTCTTTCACTCCATCGACATATCGGATTGTCCCTATACGAATAGCTTAGACAGCCG
CTTAGCCGAATTGGATTACTTACTGAATAACGATCTGGCCGATGTGGATTGCGAAAACTGGGAAGAAGACACTCCATTTAAAG
ATCCGCGCGAGCTGTATGATTTTTTAAAGACGGAAAAGCCCGAAGAGGAACTTGTCTTTTCCCACGGCGACCTGGGAGACAGC
AACATCTTTGTGAAAGATGGCAAAGTAAGTGGCTTTATTGATCTTGGGAGAAGCGGCAGGGCGGACAAGTGGTATGACATTGC
CTTCTGCGTCCGGTCGATCAGGGAGGATATCGGGGAAGAACAGTATGTCGAGCTATTTTTTGACTTACTGGGGATCAAGCCTG
ATTGGGAGAAAATAAAATATTATATTTTACTGGATGAATTGTTTTAGTACCTAGATTTAGATGTCTAAAGAATTGATGCTTT
AACTACATGCTTTTTAGACATCTAATCTTTTCTGAAGTACATCCGCAACTGTCCATACTCTGATGTTTTATATCTTTTCTAAAA
GTTCGCTAGATAGGGGTCCCGAGCGCCTACGAGGAATTTGTATCGGGGTATAGATCTAAAACGAATTTTAACAAAAAAATGTG
TTTTTCTTTGTTCTCGAGCCCGGGGCTAGCACTAGTAGACCGGGGACTTATCAGCCAACCTGTTAACCTGACTGTACAGCAAAT
CCGAAATATCAACAATCTGAAATCAGATGTGCTTTACGTTGGACAAGTATTGAAGCTGACAGGTAAAGCATCTTCAGGCTCTT
CATCATCGTCGTCTTCTTCGTCAAATGCAAGCTCCGGCACGACGACTACATACACGGTGAAAAGCGGTGATTCCTTATGGGTGA
TTGCTCAAAAATTTAATGTAACAGCTCAGCAGATTCGTGAGAAAAACAATTTAAAAACGGATGTCTTGCAGGTTGGCCAAAAG
CTGGTCATTTCCGGAAAAGCTTCATCATCGTCTTCATCCGGTTCTTCAAACACCACCAGCTCAACGAGTGCGAAGATTAACACA
ATGATTTCGGCCGCTAAAGCGCAGCTTGGGGTTCCGTATCGCTGGGGAGGCACGACACCGTCCGGGTTTGACTGCAGCGGATTC
ATTTACTATGTACTGAACAAAGTCACATCCGTATCTAGATTAACGGCAGCGGGATATTGGA

*mfd::*TnJM1

TTGAAACAGAAATTGATGTCGAGCTTGATGCGTATATTCCGGAAACTTATATTCAAGACGGCAAACAGAAAATTGATATGTAT
AAACGCTTCAGGTCTGTGGCGACAATCGAAGAGAAGAATGAGCTTCAAGATGAAATGATCGACCGCTTTGGAAACTATCCAAA
AGAAGTCGAATACTTGTTTACTGTTGCAGAAATGAAAGTCTATGCGAGACAGGAACGTGTCGAGCTGATTAAGCAGGATAAA
GATGCTGTCAGATTGACGATTTCTGAAGAAGCAAGTGCTGAAATTGACGGGCAGAAGCTGTTTGAGCTTGGCAATCAATATGG
CAGACAAATCGGACTGGGAATGGAAGGGAAAAAACTCAAAATCTCCATACAGACGAAAGGCCGTAGTGCTGATGAATGGCTCG
ACACCGTGCTGGGCATGCTGAAGGGCTTAAAAGATGTGAAAAAGCAAACCATTTCATCAACGTAAATTTTGTTACTCTCTGGT
GTAACAGGTTGGCTGATAAGTCCCCGGTCTGGTCGACTAAGTGAGGATTAACTAATAAGGAGGACAAACATGTCAAAAGGAGA
AGAACTTTTTACAGGTGTAGTACCTATCTTGGTTGAATTGGATGGTGATGTTAACGGTCACAAATTTTCTGTACGTGGTGAAG
GTGAAGGTGATGCAACTAACGGTAAATTGACACTTAAATTCATTTGTACAACTGGAAAACTTCCTGTTCCTTGGCCTACTCTT
GTTACAACATTGACATATGGAGTACAATGTTTTTCACGTTATCCTGATCATATGAAACGTCACGATTTTTTTAAATCTGCTAT
GCCAGAAGGTTATGTACAAGAACGTACAATTTCATTTAAAGATGACGGAACATATAAAACACGTGCTGAAGTAAAATTCGAA
GGTGACACTCTTGTTAATCGTATCGAATTGAAAGGAATCGATTTCAAAGAAGATGGTAACATTTTGGGACACAAACTTGAATA
CAACTTCAACTCTCATAATGTTTATATCACAGCTGACAAACAAAAAAACGGTATTAAAGCTAATTTTAAAATTCGTCACAATG
TTGAAGATGGATCTGTTCAATTGGCTGATCATTATCAACAAAATACACCAATCGGAGACGGACCAGTATTGCTTCCAGATAAC
CACTACCTTTCTACTCAATCAGTTCTTTCAAAAGATCCTAACGAAAAACGTGACCATATGGTACTTCTTGAATTTGTTACAGC
AGCAGGTATCACTCACGGTATGGACGAACTTTATAAATAAGGATCCTACTCGAGCCACATAGATGGCGTCGCTAGTATTAAAT
GCATATTATTTTTATATAGTACCAACCTTCAAATGATTCCCTATAGCTTGTAAATTCTATCATAATTGTGGTTTCAAAATCGG
CTCCGTCGATACTATGTTATACGCCAACTTTGAAAACAACTTTGAAAAAGCTGTTTTCTGGTATTTAAGGTTTTAGAATGCAA
GGAACAGTGAATTGGAGTTCGTCTTGTTATAATTAGCTTCTTGGGGTATCTTTAAATACTGTAGAAAAGAGGAAGGAAATAA

TAAATGGCTAAAATGAGAATATCACCGGAATTGAAAAAACTGATCGAAAAATACCGCTGCGTAAAAGATACGGAAGGAATGT
CTCCTGCTAAGGTATATAAGCTGGTGGGAGAAAATGAAAACCTATATTTAAAAATGACGGACAGCCGGTATAAAGGGACCACC
TATGATGTAGAACGGGAAAAGGACATGATGCTATGGCTGGAAGGAAAGCTGCCTGTTCCAAAGGTCCTGCACTTTGAACGGCA
TGATGGCTGGAGCAATCTGCTCATGAGTGAGGCCGATGGCGTCCTTTGCTCGGAAGAGTATGAAGATGAACAAAGCCCTGAAA
AGATTATCGAGCTGTATGCGGAGTGCATCAGGCTCTTTCACTCCATCGACATATCGGATTGTCCCTATACGAATAGCTTAGAC
AGCCGCTTAGCCGAATTGGATTACTTACTGAATAACGATCTGGCCGATGTGGATTGCGAAAACTGGGAAGAAGACACTCCATT
TAAAGATCCGCGCGAGCTGTATGATTTTTTAAAGACGGAAAAGCCCGAAGAGGAACTTGTCTTTTCCCACGGCGACCTGGGAG
ACAGCAACATCTTTGTGAAAGATGGCAAAGTAAGTGGCTTTATTGATCTTGGGAGAAGCGGCAGGGCGGACAAGTGGTATGAC
ATTGCCTTCTGCGTCCGGTCGATCAGGGAGGATATCGGGGAAGAACAGTATGTCGAGCTATTTTTTGACTTACTGGGGATCAA
GCCTGATTGGGAGAAAATAAAATATTATATTTTACTGGATGAATTGTTTTAGTACCTAGATTTAGATGTCTAAAGAATTGAT
GCTTTAACTACATGCTTTTTAGACATCTAATCTTTTCTGAAGTACATCCGCAACTGTCCATACTCTGATGTTTTATATCTTTTC
TAAAAGTTCGCTAGATAGGGGTCCCGAGCGCCTACGAGGAATTTGTATCGGGGTATAGATCTAAAACGAATTTTAACAAAAAA
ATGTGTTTTTCTTTGTTCTCGAGCCCGGGGCTAGCACTAGTAGACCGGGGACTTATCAGCCAACCTGTTATTACATTTGATGTG
ACGGATACTAATTTCAAGCGAGGCGGAAGGTACATAA

*msmX::*TnJM1

TGCGAATGGTCGCAGGACTTGAAGAAATTTCGAAAGGTGATTTTTATATTGAAGGAAAACGGGTCAATGATGTAGCGCCAAA
GGACAGGGATATCGCGATGGTATTTCAGAACTACGCGCTTTATCCGCATATGACGGTCTACGATAATATCGCGTTCGGGCTCA
AGCTTCGGAAAATGCCGAAGCCTGAAATCAAAAAAAGAGTCGAAGAAGCCGCTAAAATTCTCGGGCTTGAGGAATATTTGCAC
CGTAAACCGAAAGCGCTGTCAGGCGGACAGAGACAGCGGGTTGCGCTGGGCCGGGCAATCGTGCGGGATGCAAAGGTGTTCCT
GATGGATGAGCCTTTGTCAAACCTGGACGCGAAGCTGAGGGTGCAAATGCGGGCGGAAATCATTAAGCTCCACCAGAGATTGC
AGACTACAACGATTTATGTGACGCATGACCAGACAGAAGCGCTGACAATGGCGACACGGATTGTAGTCATGAAAGATGGGAAA
ATTCAGCAGATCGGGACGCCGAAGGATGTAACAGGTTGGCTGATAAGTCCCCGGTCTGGTCGACTAAGTGAGGATTAACTAAT
AAGGAGGACAAACATGTCAAAAGGAGAAGAACTTTTTACAGGTGTAGTACCTATCTTGGTTGAATTGGATGGTGATGTTAAC
GGTCACAAATTTTCTGTACGTGGTGAAGGTGAAGGTGATGCAACTAACGGTAAATTGACACTTAAATTCATTTGTACAACTGG
AAAACTTCCTGTTCCTTGGCCTACTCTTGTTACAACATTGACATATGGAGTACAATGTTTTTCACGTTATCCTGATCATATGA
AACGTCACGATTTTTTTAAATCTGCTATGCCAGAAGGTTATGTACAAGAACGTACAATTTCATTTAAAGATGACGGAACATAT
AAAACACGTGCTGAAGTAAAATTCGAAGGTGACACTCTTGTTAATCGTATCGAATTGAAAGGAATCGATTTCAAAGAAGATG
GTAACATTTTGGGACACAAACTTGAATACAACTTCAACTCTCATAATGTTTATATCACAGCTGACAAACAAAAAAACGGTATT
AAAGCTAATTTTAAAATTCGTCACAATGTTGAAGATGGATCTGTTCAATTGGCTGATCATTATCAACAAAATACACCAATCGG
AGACGGACCAGTATTGCTTCCAGATAACCACTACCTTTCTACTCAATCAGTTCTTTCAAAAGATCCTAACGAAAAACGTGACC
ATATGGTACTTCTTGAATTTGTTACAGCAGCAGGTATCACTCACGGTATGGACGAACTTTATAAATAAGGATCCTACTCGAGC
CACATAGATGGCGTCGCTAGTATTAAATGCATATTATTTTTATATAGTACCAACCTTCAAATGATTCCCTATAGCTTGTAAAT
TCTATCATAATTGTGGTTTCAAAATCGGCTCCGTCGATACTATGTTATACGCCAACTTTGAAAACAACTTTGAAAAAGCTGTT
TTCTGGTATTTAAGGTTTTAGAATGCAAGGAACAGTGAATTGGAGTTCGTCTTGTTATAATTAGCTTCTTGGGGTATCTTTAA
ATACTGTAGAAAAGAGGAAGGAAATAATAAATGGCTAAAATGAGAATATCACCGGAATTGAAAAAACTGATCGAAAAATACC
GCTGCGTAAAAGATACGGAAGGAATGTCTCCTGCTAAGGTATATAAGCTGGTGGGAGAAAATGAAAACCTATATTTAAAAAT
GACGGACAGCCGGTATAAAGGGACCACCTATGATGTAGAACGGGAAAAGGACATGATGCTATGGCTGGAAGGAAAGCTGCCTG
TTCCAAAGGTCCTGCACTTTGAACGGCATGATGGCTGGAGCAATCTGCTCATGAGTGAGGCCGATGGCGTCCTTTGCTCGGAA
GAGTATGAAGATGAACAAAGCCCTGAAAAGATTATCGAGCTGTATGCGGAGTGCATCAGGCTCTTTCACTCCATCGACATATC
GGATTGTCCCTATACGAATAGCTTAGACAGCCGCTTAGCCGAATTGGATTACTTACTGAATAACGATCTGGCCGATGTGGATT
GCGAAAACTGGGAAGAAGACACTCCATTTAAAGATCCGCGCGAGCTGTATGATTTTTTAAAGACGGAAAAGCCCGAAGAGGAA
CTTGTCTTTTCCCACGGCGACCTGGGAGACAGCAACATCTTTGTGAAAGATGGCAAAGTAAGTGGCTTTATTGATCTTGGGAG
AAGCGGCAGGGCGGACAAGTGGTATGACATTGCCTTCTGCGTCCGGTCGATCAGGGAGGATATCGGGGAAGAACAGTATGTCG
AGCTATTTTTTGACTTACTGGGGATCAAGCCTGATTGGGAGAAAATAAAATATTATATTTTACTGGATGAATTGTTTTAGTAC
CTAGATTTAGATGTCTAAAGAATTGATGCTTTAACTACATGCTTTTTAGACATCTAATCTTTTCTGAAGTACATCCGCAACTG
TCCATACTCTGATGTTTTATATCTTTTCTAAAAGTTCGCTAGATAGGGGTCCCGAGCGCCTACGAGGAATTTGTATCGGGGTA
TAGATCTAAAACGAATTTTAACAAAAAAATGTGTTTTTCTTTGTTCTCGAGCCCGGGGCTAGCACTAGTAGACCGGGGACTTA
TCAGCCAACCTGTTATATGAATTCCCTGAAAACGTCTTTGTCGGCGGGTTTATCGGATCACCGGCGATGAATTTTTTCAAAGG
AAAGCTCACGGATGGCTTAATCAAAATCGGTTCTGCGGCATTAACCGTCCCGGAAGGAAAAATGAAAGTGCTGCGTGAAAAAG
GCTACATCGGCAAAGAGGTCATCTTCGGCATCCGTCCTGAGGATATACATGATGAATTGATTGTCGTGGAATCATATAAGAAC
TCTTCGATTAAGGCGAAAATTAATGTTGCAGAGCTGCTCGGTTCTGAAATTATGATTTATTCGCAGATAGACAACCAAGACTT
TATTGCGCGGATCGACGCCCGCCTCGATATTCAATCGGGCGATGAGCTGACGGTTGCATTTGATATGAATAAAGGCCATTTCT
TTGACAGTGAGACAGAAGTGAGAATCCGATAAGATCAAAAAAACCGGACATGGAGACATGTCCGGTTTTTTGCTATTG

*pucF::*TnJM1

CATATTGAACAGGGGAAGACGTTGGAAATGTCAGGCCGGGATCTCGGCATCGTGACAAGTATTGCGGGGCAGAGACGATATCT
CGTCACGCTCGAAGGAGAATGCAATCACGCAGGAACCACCTCCATGAAATGGCGCAAGGACCCGCTCGCAGCCAGCAGCCGTAT
CATTCATGAGCTGCTGCTGCGGTCGGATGAGCTGCCGGATGAGCTCCGTCTGACATGCGGAAAAATAACGGCAGAGCCCAATG
TAGCCAATGTCATACCGGGCCGCGTCCAGTTTTCAATCGATATTCGCCATCAGCATCAGCATGTGCTGGAACAGTTTCATCAAG
ACATGGTTGCTTTGATCAACGGCATTTGCCTACAAAAAGGAATTCGCGCTGTGATTGATGAATATATGCGGATAGAGCCTGTG
CCGATGGACGAAAGGCTGAAGGCTGCGGCTTTTGAAACAGCATTAGAAAACGGCTTCAGCTGTGAGGAAATGGTGAGCGGAGC
AGGGCATGACGCGCAAATGATCGGAAGGCGCTATCCTGCTTGTAACAGGTTGGCTGATAAGTCCCCGGTCTGGTCGACTAAGT
GAGGATTAACTAATAAGGAGGACAAACATGTCAAAAGGAGAAGAACTTTTTACAGGTGTAGTACCTATCTTGGTTGAATTGG
ATGGTGATGTTAACGGTCACAAATTTTCTGTACGTGGTGAAGGTGAAGGTGATGCAACTAACGGTAAATTGACACTTAAATTC
ATTTGTACAACTGGAAAACTTCCTGTTCCTTGGCCTACTCTTGTTACAACATTGACATATGGAGTACAATGTTTTTCACGTTA
TCCTGATCATATGAAACGTCACGATTTTTTTAAATCTGCTATGCCAGAAGGTTATGTACAAGAACGTACAATTTCATTTAAAG
ATGACGGAACATATAAAACACGTGCTGAAGTAAAATTCGAAGGTGACACTCTTGTTAATCGTATCGAATTGAAAGGAATCGAT
TTCAAAGAAGATGGTAACATTTTGGGACACAAACTTGAATACAACTTCAACTCTCATAATGTTTATATCACAGCTGACAAACA
AAAAAACGGTATTAAAGCTAATTTTAAAATTCGTCACAATGTTGAAGATGGATCTGTTCAATTGGCTGATCATTATCAACAAA
ATACACCAATCGGAGACGGACCAGTATTGCTTCCAGATAACCACTACCTTTCTACTCAATCAGTTCTTTCAAAAGATCCTAACG
AAAAACGTGACCATATGGTACTTCTTGAATTTGTTACAGCAGCAGGTATCACTCACGGTATGGACGAACTTTATAAATAAGGA
TCCTACTCGAGCCACATAGATGGCGTCGCTAGTATTAAATGCATATTATTTTTATATAGTACCAACCTTCAAATGATTCCCTA
TAGCTTGTAAATTCTATCATAATTGTGGTTTCAAAATCGGCTCCGTCGATACTATGTTATACGCCAACTTTGAAAACAACTTT
GAAAAAGCTGTTTTCTGGTATTTAAGGTTTTAGAATGCAAGGAACAGTGAATTGGAGTTCGTCTTGTTATAATTAGCTTCTTG
GGGTATCTTTAAATACTGTAGAAAAGAGGAAGGAAATAATAAATGGCTAAAATGAGAATATCACCGGAATTGAAAAAACTGA
TCGAAAAATACCGCTGCGTAAAAGATACGGAAGGAATGTCTCCTGCTAAGGTATATAAGCTGGTGGGAGAAAATGAAAACCT
ATATTTAAAAATGACGGACAGCCGGTATAAAGGGACCACCTATGATGTAGAACGGGAAAAGGACATGATGCTATGGCTGGAA
GGAAAGCTGCCTGTTCCAAAGGTCCTGCACTTTGAACGGCATGATGGCTGGAGCAATCTGCTCATGAGTGAGGCCGATGGCGT
CCTTTGCTCGGAAGAGTATGAAGATGAACAAAGCCCTGAAAAGATTATCGAGCTGTATGCGGAGTGCATCAGGCTCTTTCACT
CCATCGACATATCGGATTGTCCCTATACGAATAGCTTAGACAGCCGCTTAGCCGAATTGGATTACTTACTGAATAACGATCTG
GCCGATGTGGATTGCGAAAACTGGGAAGAAGACACTCCATTTAAAGATCCGCGCGAGCTGTATGATTTTTTAAAGACGGAAAA
GCCCGAAGAGGAACTTGTCTTTTCCCACGGCGACCTGGGAGACAGCAACATCTTTGTGAAAGATGGCAAAGTAAGTGGCTTTA
TTGATCTTGGGAGAAGCGGCAGGGCGGACAAGTGGTATGACATTGCCTTCTGCGTCCGGTCGATCAGGGAGGATATCGGGGAA
GAACAGTATGTCGAGCTATTTTTTGACTTACTGGGGATCAAGCCTGATTGGGAGAAAATAAAATATTATATTTTACTGGATGA
ATTGTTTTAGTACCTAGATTTAGATGTCTAAAGAATTGATGCTTTAACTACATGCTTTTTAGACATCTAATCTTTTCTGAAGT
ACATCCGCAACTGTCCATACTCTGATGTTTTATATCTTTTCTAAAAGTTCGCTAGATAGGGGTCCCGAGCGCCTACGAGGAATT
TGTATCGGGGTATAGATCTAAAACGAATTTTAACAAAAAAATGTGTTTTTCTTTGTTCTCGAGCCCGGGGCTAGCACTAGTAG
ACCGGGGACTTATCAGCCAACCTGTTGTATGCTGTTTGTGCCGAGCCGAGGCGGCGTCAGCCACTCACCGAAGGAATATACGTC
AGCCAGACAGCTTGAGATCGGCGTCCGCGCACTGACTGATTTATTATACAAACTGGCTTACTGATAAAGGAGGAATGGCTGTG
TCAGGCAGAAGAGAATTATGCACCCCGTTAAGAACGATTATGACACCGGGGCCTGTTGAGGTTGATCCGCGTGTATTAAGAGT
AATGAGCACCCCGGTTGTCGGCCAATTTGATCCGGCGTTTACAGGTATTATGAATGAAACGATGGAGATGCTTCGGGAGCTGT
TCCAAACAAAAAACCGCTGGGCATACCCGATTGACGGCACTTCACGGGCGGGAATTGAAGCAGTGCTGGCTAGTGTGATAGAG
CCGGAAGATGATGTGCTCATTCCCATTTACGGCCGTTTCGGTTATTTGCTGACTGAAATCGCCGAGCGATATGGGGCAAATGT
TCATATGCTGGAGTGCGAATGGG

*rapD::*TnJM1

GAATTAATCTCTTTTTCAACCGCGCTAAAATGTAACCAACTGTCAATGAGAGCCGTCAAAAGTTATGATATGATAATTATAGA
TTTTACCAATAGCAAAGAGGTAGGAAAATGATTTCGAAGTTTATTGAAAAAAGAATGCTAAACATGTTAGACGAGTGGTATT
CCGCAATGAGCAAACGCAAAATGAATCACGTTTGCACACTGAAAGAGAAAATTGATCAGCACCTTCCAAAGATCAAAAAGAAC
ACAAAACTTTGGATGCGTTATCAATTGTTTCAGGCCCGCCATCAGCTGCTTTTTGAAAACCAGAACGGACTCGATTCATTGTTC
GATGGCCTATATGGCCTGGAAGACAAAATGGATGATGAATTGAAATATTATCTGTATTTTTTCTCTGGGTTATATGAGATGGT
AAAAACAGCTCCGAAACATGCAGTAACAGGTTGGCTGATAAGTCCCCGGTCTGGTCGACTAAGTGAGGATTAACTAATAAGGA
GGACAAACATGTCAAAAGGAGAAGAACTTTTTACAGGTGTAGTACCTATCTTGGTTGAATTGGATGGTGATGTTAACGGTCAC
AAATTTTCTGTACGTGGTGAAGGTGAAGGTGATGCAACTAACGGTAAATTGACACTTAAATTCATTTGTACAACTGGAAAACT
TCCTGTTCCTTGGCCTACTCTTGTTACAACATTGACATATGGAGTACAATGTTTTTCACGTTATCCTGATCATATGAAACGTCA
CGATTTTTTTAAATCTGCTATGCCAGAAGGTTATGTACAAGAACGTACAATTTCATTTAAAGATGACGGAACATATAAAACAC
GTGCTGAAGTAAAATTCGAAGGTGACACTCTTGTTAATCGTATCGAATTGAAAGGAATCGATTTCAAAGAAGATGGTAACAT
TTTGGGACACAAACTTGAATACAACTTCAACTCTCATAATGTTTATATCACAGCTGACAAACAAAAAAACGGTATTAAAGCTA
ATTTTAAAATTCGTCACAATGTTGAAGATGGATCTGTTCAATTGGCTGATCATTATCAACAAAATACACCAATCGGAGACGGA
CCAGTATTGCTTCCAGATAACCACTACCTTTCTACTCAATCAGTTCTTTCAAAAGATCCTAACGAAAAACGTGACCATATGGT
ACTTCTTGAATTTGTTACAGCAGCAGGTATCACTCACGGTATGGACGAACTTTATAAATAAGGATCCTACTCGAGCCACATAG
ATGGCGTCGCTAGTATTAAATGCATATTATTTTTATATAGTACCAACCTTCAAATGATTCCCTATAGCTTGTAAATTCTATCA
TAATTGTGGTTTCAAAATCGGCTCCGTCGATACTATGTTATACGCCAACTTTGAAAACAACTTTGAAAAAGCTGTTTTCTGGT
ATTTAAGGTTTTAGAATGCAAGGAACAGTGAATTGGAGTTCGTCTTGTTATAATTAGCTTCTTGGGGTATCTTTAAATACTGT

AGAAAAGAGGAAGGAAATAATAAATGGCTAAAATGAGAATATCACCGGAATTGAAAAAACTGATCGAAAAATACCGCTGCGT
AAAAGATACGGAAGGAATGTCTCCTGCTAAGGTATATAAGCTGGTGGGAGAAAATGAAAACCTATATTTAAAAATGACGGAC
AGCCGGTATAAAGGGACCACCTATGATGTAGAACGGGAAAAGGACATGATGCTATGGCTGGAAGGAAAGCTGCCTGTTCCAAA
GGTCCTGCACTTTGAACGGCATGATGGCTGGAGCAATCTGCTCATGAGTGAGGCCGATGGCGTCCTTTGCTCGGAAGAGTATG
AAGATGAACAAAGCCCTGAAAAGATTATCGAGCTGTATGCGGAGTGCATCAGGCTCTTTCACTCCATCGACATATCGGATTGT
CCCTATACGAATAGCTTAGACAGCCGCTTAGCCGAATTGGATTACTTACTGAATAACGATCTGGCCGATGTGGATTGCGAAAA
CTGGGAAGAAGACACTCCATTTAAAGATCCGCGCGAGCTGTATGATTTTTTAAAGACGGAAAAGCCCGAAGAGGAACTTGTCT
TTTCCCACGGCGACCTGGGAGACAGCAACATCTTTGTGAAAGATGGCAAAGTAAGTGGCTTTATTGATCTTGGGAGAAGCGGC
AGGGCGGACAAGTGGTATGACATTGCCTTCTGCGTCCGGTCGATCAGGGAGGATATCGGGGAAGAACAGTATGTCGAGCTATT
TTTTGACTTACTGGGGATCAAGCCTGATTGGGAGAAAATAAAATATTATATTTTACTGGATGAATTGTTTTAGTACCTAGATT
TAGATGTCTAAAGAATTGATGCTTTAACTACATGCTTTTTAGACATCTAATCTTTTCTGAAGTACATCCGCAACTGTCCATAC
TCTGATGTTTTATATCTTTTCTAAAAGTTCGCTAGATAGGGGTCCCGAGCGCCTACGAGGAATTTGTATCGGGGTATAGATCT
AAAACGAATTTTAACAAAAAAATGTGTTTTTCTTTGTTCTCGAGCCCGGGGCTAGCACTAGTAGACCGGGGACTTATCAGCCA
ACCTGTTACATCATTTTAAAAAAGCTGAACAGTACCTGGCCGCCATTCACAATACGTTTGAAGCCGCTGATTTATATTATCAA
ACCGCCGGCGCCTATTACTTGATGAAATCACCGCCGCTTTCCGTCCAATACGTCAAAAAAGCATTACACATCTATTTGCACCAA
TTCGGCTATATCAAAAAGGTAATCACCTGTAAACTTTTGCTCGCCGTGAATTATATTGATCAAGAACGCTATGAAAAAGCTGA
ACAGCTTTTCAAAGAAATCATTAAGAAAACCCAGCAACTACATGACGAAAACCTGCTATGCCACGCTTATTATAACCTTGGCT
TTTTAAAAGCGACCGAGAAAAAAGACCAGGAAGCGCTTCTTTACTTTAGAAAAGTATTAAAGAATCAAGAATTTGAAATG

*sdpB::*TnJM1

TTCCTATTATTTTCTTCTTATTACTTTAGCAATATTTCACCTCAGAATCCACTGTTCAAAAAAAATTTTTTGCAACAATTGTCT
CCCCAAGGCTTTGGCTTTTATAGTAAAAGCCCTACAGAAGAAAACATTTCATTTCACACAAAAGAAAATTTAAAGTTACCTAA
TGCACTTCCCAATAATTTTTTTGGGATAAAAAGAGAAGGAAGAGTTCAGGCAATAGAATTAGGCAAAATTGTAGAGAATATC
GATCCAAAGAATTGGAAAACTTGTGAAAACAACAACTCCTGCACAAATTTAGAGAAACAAATAAAGCCTATTAAGGTTATAA
AAAATGAAGATTATATACATCTTAGCAAAGGAGAATACCTAATATATCGCCAAAAACCACTCTCATGGTATTGGATAGACTTT
AAGCAAACTACCTCTTTTGAAAGAAAGGTGCTAAAAATAAAAATAGTATGAAGATATTAAATAGTTTAGAAGGTTATATTGA
CACCTAACAGGTTGGCTGATAAGTCCCCGGTCTGGTCGACTAAGTGAGGATTAACTAATAAGGAGGACAAACATGTCAAAAGG
AGAAGAACTTTTTACAGGTGTAGTACCTATCTTGGTTGAATTGGATGGTGATGTTAACGGTCACAAATTTTCTGTACGTGGTG
AAGGTGAAGGTGATGCAACTAACGGTAAATTGACACTTAAATTCATTTGTACAACTGGAAAACTTCCTGTTCCTTGGCCTACT
CTTGTTACAACATTGACATATGGAGTACAATGTTTTTCACGTTATCCTGATCATATGAAACGTCACGATTTTTTTAAATCTGC
TATGCCAGAAGGTTATGTACAAGAACGTACAATTTCATTTAAAGATGACGGAACATATAAAACACGTGCTGAAGTAAAATTC
GAAGGTGACACTCTTGTTAATCGTATCGAATTGAAAGGAATCGATTTCAAAGAAGATGGTAACATTTTGGGACACAAACTTGA
ATACAACTTCAACTCTCATAATGTTTATATCACAGCTGACAAACAAAAAAACGGTATTAAAGCTAATTTTAAAATTCGTCACA
ATGTTGAAGATGGATCTGTTCAATTGGCTGATCATTATCAACAAAATACACCAATCGGAGACGGACCAGTATTGCTTCCAGAT
AACCACTACCTTTCTACTCAATCAGTTCTTTCAAAAGATCCTAACGAAAAACGTGACCATATGGTACTTCTTGAATTTGTTAC
AGCAGCAGGTATCACTCACGGTATGGACGAACTTTATAAATAAGGATCCTACTCGAGCCACATAGATGGCGTCGCTAGTATTA
AATGCATATTATTTTTATATAGTACCAACCTTCAAATGATTCCCTATAGCTTGTAAATTCTATCATAATTGTGGTTTCAAAAT
CGGCTCCGTCGATACTATGTTATACGCCAACTTTGAAAACAACTTTGAAAAAGCTGTTTTCTGGTATTTAAGGTTTTAGAATG
CAAGGAACAGTGAATTGGAGTTCGTCTTGTTATAATTAGCTTCTTGGGGTATCTTTAAATACTGTAGAAAAGAGGAAGGAAA
TAATAAATGGCTAAAATGAGAATATCACCGGAATTGAAAAAACTGATCGAAAAATACCGCTGCGTAAAAGATACGGAAGGAA
TGTCTCCTGCTAAGGTATATAAGCTGGTGGGAGAAAATGAAAACCTATATTTAAAAATGACGGACAGCCGGTATAAAGGGACC
ACCTATGATGTAGAACGGGAAAAGGACATGATGCTATGGCTGGAAGGAAAGCTGCCTGTTCCAAAGGTCCTGCACTTTGAACG
GCATGATGGCTGGAGCAATCTGCTCATGAGTGAGGCCGATGGCGTCCTTTGCTCGGAAGAGTATGAAGATGAACAAAGCCCTG
AAAAGATTATCGAGCTGTATGCGGAGTGCATCAGGCTCTTTCACTCCATCGACATATCGGATTGTCCCTATACGAATAGCTTA
GACAGCCGCTTAGCCGAATTGGATTACTTACTGAATAACGATCTGGCCGATGTGGATTGCGAAAACTGGGAAGAAGACACTCC
ATTTAAAGATCCGCGCGAGCTGTATGATTTTTTAAAGACGGAAAAGCCCGAAGAGGAACTTGTCTTTTCCCACGGCGACCTGG
GAGACAGCAACATCTTTGTGAAAGATGGCAAAGTAAGTGGCTTTATTGATCTTGGGAGAAGCGGCAGGGCGGACAAGTGGTAT
GACATTGCCTTCTGCGTCCGGTCGATCAGGGAGGATATCGGGGAAGAACAGTATGTCGAGCTATTTTTTGACTTACTGGGGAT
CAAGCCTGATTGGGAGAAAATAAAATATTATATTTTACTGGATGAATTGTTTTAGTACCTAGATTTAGATGTCTAAAGAATT
GATGCTTTAACTACATGCTTTTTAGACATCTAATCTTTTCTGAAGTACATCCGCAACTGTCCATACTCTGATGTTTTATATCTT
TTCTAAAAGTTCGCTAGATAGGGGTCCCGAGCGCCTACGAGGAATTTGTATCGGGGTATAGATCTAAAACGAATTTTAACAAA
AAAATGTGTTTTTCTTTGTTCTCGAGCCCGGGGCTAGCACTAGTAGACCGGGGACTTATCAGCCAACCTGTTATAATCCATGG
AAAAATACATATGCACTTTTTAGAAGTTTACTTGGTTTCTCAACATTACTAGTACTATTATTCAATAGTACTGATATTTTATT
TAGTTATAGTGCAAATAATGTCACATGTGAAAATGTCTATATCCCTACCGCTTTTTGTTTTGCTAAAGAATATAGTATCAATT
TTGAGATTATAAGATACTTAATGATTTTTATATTAACCTTAGTGGTTATAGGGTGGAGACCTAGATTTACCGGTTTATTTCAC
TGGTATATTTGCTATAGTATTCAAACTTCAGCTTTAACTATCGATGGTGGAGAGCAAATTGCAACTGTTCTTTCTTTTCTTAT
ATTACCTGTTACATTATTAGATTCAAGGCGAAATCATTGGAATATAAAGAAAAACAATAATGAATCTTTCACAAAGAAGACA
GTATTGTTTTATATAATGACAATAATTAAAATTCAAGTTTTTATCATTTATTTAAACGCAGCTTTAGAGCGA

*wapA::*TnJM*1*

AAAAGCACAAGCACCGTCAGCAAAGGGCTACAATAATGGGAATGCCACAGGATACTTCGACCTTTCTTGGAAAGCTGTATCCG
GTGCAACCGGCTATAAGGTTCAGGTGTTCAATGGGAAAGGCTTTGAGACACTTGATCTCGGAAATCAGACGTCTTGGACCACA
AAAGGGAAAAAGATCTGGCCGACAAGTGCAGAAATCAAGGCGGGAAAATATGCTCTTCACCTAAAGGACGGAAGCGGAGCAG
AGCTGCCGATCAATCCCGGACCAACCTATAAAAACGCCGGGGGAGATGGAGCCAAAAGAAATTACTCGTTTAAAATTATCGCA
TACAACAAAGACGGCGAAGCCATTGCATCCCCGGCTGCTACCCCGGCACTTCCTGATATCGCTAGGCCAAAGAATGTAACTGGC
TATTTGTATAACAGGTTGGCTGATAAGTCCCCGGTCTGGTCGACTAAGTGAGGATTAACTAATAAGGAGGACAAACATGTCAA
AAGGAGAAGAACTTTTTACAGGTGTAGTACCTATCTTGGTTGAATTGGATGGTGATGTTAACGGTCACAAATTTTCTGTACGT
GGTGAAGGTGAAGGTGATGCAACTAACGGTAAATTGACACTTAAATTCATTTGTACAACTGGAAAACTTCCTGTTCCTTGGCC
TACTCTTGTTACAACATTGACATATGGAGTACAATGTTTTTCACGTTATCCTGATCATATGAAACGTCACGATTTTTTTAAAT
CTGCTATGCCAGAAGGTTATGTACAAGAACGTACAATTTCATTTAAAGATGACGGAACATATAAAACACGTGCTGAAGTAAAA
TTCGAAGGTGACACTCTTGTTAATCGTATCGAATTGAAAGGAATCGATTTCAAAGAAGATGGTAACATTTTGGGACACAAACT
TGAATACAACTTCAACTCTCATAATGTTTATATCACAGCTGACAAACAAAAAAACGGTATTAAAGCTAATTTTAAAATTCGTC
ACAATGTTGAAGATGGATCTGTTCAATTGGCTGATCATTATCAACAAAATACACCAATCGGAGACGGACCAGTATTGCTTCCA
GATAACCACTACCTTTCTACTCAATCAGTTCTTTCAAAAGATCCTAACGAAAAACGTGACCATATGGTACTTCTTGAATTTGT
TACAGCAGCAGGTATCACTCACGGTATGGACGAACTTTATAAATAAGGATCCTACTCGAGCCACATAGATGGCGTCGCTAGTA
TTAAATGCATATTATTTTTATATAGTACCAACCTTCAAATGATTCCCTATAGCTTGTAAATTCTATCATAATTGTGGTTTCAA
AATCGGCTCCGTCGATACTATGTTATACGCCAACTTTGAAAACAACTTTGAAAAAGCTGTTTTCTGGTATTTAAGGTTTTAGA
ATGCAAGGAACAGTGAATTGGAGTTCGTCTTGTTATAATTAGCTTCTTGGGGTATCTTTAAATACTGTAGAAAAGAGGAAGG
AAATAATAAATGGCTAAAATGAGAATATCACCGGAATTGAAAAAACTGATCGAAAAATACCGCTGCGTAAAAGATACGGAAG
GAATGTCTCCTGCTAAGGTATATAAGCTGGTGGGAGAAAATGAAAACCTATATTTAAAAATGACGGACAGCCGGTATAAAGG
GACCACCTATGATGTAGAACGGGAAAAGGACATGATGCTATGGCTGGAAGGAAAGCTGCCTGTTCCAAAGGTCCTGCACTTTG
AACGGCATGATGGCTGGAGCAATCTGCTCATGAGTGAGGCCGATGGCGTCCTTTGCTCGGAAGAGTATGAAGATGAACAAAGC
CCTGAAAAGATTATCGAGCTGTATGCGGAGTGCATCAGGCTCTTTCACTCCATCGACATATCGGATTGTCCCTATACGAATAG
CTTAGACAGCCGCTTAGCCGAATTGGATTACTTACTGAATAACGATCTGGCCGATGTGGATTGCGAAAACTGGGAAGAAGACA
CTCCATTTAAAGATCCGCGCGAGCTGTATGATTTTTTAAAGACGGAAAAGCCCGAAGAGGAACTTGTCTTTTCCCACGGCGAC
CTGGGAGACAGCAACATCTTTGTGAAAGATGGCAAAGTAAGTGGCTTTATTGATCTTGGGAGAAGCGGCAGGGCGGACAAGTG
GTATGACATTGCCTTCTGCGTCCGGTCGATCAGGGAGGATATCGGGGAAGAACAGTATGTCGAGCTATTTTTTGACTTACTGG
GGATCAAGCCTGATTGGGAGAAAATAAAATATTATATTTTACTGGATGAATTGTTTTAGTACCTAGATTTAGATGTCTAAAG
AATTGATGCTTTAACTACATGCTTTTTAGACATCTAATCTTTTCTGAAGTACATCCGCAACTGTCCATACTCTGATGTTTTAT
ATCTTTTCTAAAAGTTCGCTAGATAGGGGTCCCGAGCGCCTACGAGGAATTTGTATCGGGGTATAGATCTAAAACGAATTTTA
ACAAAAAAATGTGTTTTTCTTTGTTCTCGAGCCCGGGGCTAGCACTAGTAGACCGGGGACTTATCAGCCAACCTGTTACAAAT
ACAAAATCGAGTCAAACGGGTTATGTGAATTTGATATGGGAAAAGGTTCAAAATGCGAAGGGCTATAAAGTTAACATTTACA
ACGGTAAAGAATATCAGTCCTTTGATGTTGGTGATGCTGATCATTGGACAACCCAAAATAAAAACATCTGGCCGACTTCTGAG
GAAATCAAAGCCGGAAGCTATAAGCTTCATACAGATGGAAAAGGCGGGGAATTAGCCCTCGATCCATCACCTGTCTATAACAA
TGCAAATGGGAATTACAAAGGGAAGAAGAATTATTCTTTCACGCTTGTCGCCTATGATGCGAATGGTGAAACGATTCCAACGG
CGCCCTTTAACCCAACGTTCCATGAAGGCGCAGAATTCCTAGGTACAGAAGAGTACTGGTCTATCATTGATATCCCAAGCGGCC
AGTTAAATGGTGCGACAGGCAATGTCATTGTGAATGA

*yaaH::*TnJM1

CGATTCCTAAGGACTGTATCGCGTCAAACAGTGACGAAGATAACGAATTCGCCTTAACCATGATGGAGAATGTTCTTTTCGCA
GAAATTACGACGGAAGAACAAATTATAGAAAAATAAACATGATCAGCGCTTTTCTTTCATACATTGATAGCGATATGAAAGG
AGGCGTTTTTCATTCAAATTTATGTGGTAAAACAAGGCGACACTCTTTCTGCTATCGCTTCACAATACAGAACAACCACAAAT
GACATCACTGAAACGAATGAAATACCGAATCCCGACAGCCTTGTTGTCGGACAAACCATTGTCATTCCAATAGCTGGCCAGTT
CTATGATGTGAAGCGAGGTGATACCCTGACATCCATCGCCCGGCAGTTCAATACAACAGCAGCCGAGCTCGCAAGGGTTAACC
GCATCCAGTTAAATACCGTGCTTCAGATTGGTTTCCGTTTATACATCCCTCCAGCTCCTAAACGAGACATCGAATCAAATGCTT
AACAGGTTGGCTGATAAGTCCCCGGTCTGGTCGACTAAGTGAGGATTAACTAATAAGGAGGACAAACATGTCAAAAGGAGAA
GAACTTTTTACAGGTGTAGTACCTATCTTGGTTGAATTGGATGGTGATGTTAACGGTCACAAATTTTCTGTACGTGGTGAAGG
TGAAGGTGATGCAACTAACGGTAAATTGACACTTAAATTCATTTGTACAACTGGAAAACTTCCTGTTCCTTGGCCTACTCTTG
TTACAACATTGACATATGGAGTACAATGTTTTTCACGTTATCCTGATCATATGAAACGTCACGATTTTTTTAAATCTGCTATG
CCAGAAGGTTATGTACAAGAACGTACAATTTCATTTAAAGATGACGGAACATATAAAACACGTGCTGAAGTAAAATTCGAAG
GTGACACTCTTGTTAATCGTATCGAATTGAAAGGAATCGATTTCAAAGAAGATGGTAACATTTTGGGACACAAACTTGAATAC
AACTTCAACTCTCATAATGTTTATATCACAGCTGACAAACAAAAAAACGGTATTAAAGCTAATTTTAAAATTCGTCACAATGT
TGAAGATGGATCTGTTCAATTGGCTGATCATTATCAACAAAATACACCAATCGGAGACGGACCAGTATTGCTTCCAGATAACC
ACTACCTTTCTACTCAATCAGTTCTTTCAAAAGATCCTAACGAAAAACGTGACCATATGGTACTTCTTGAATTTGTTACAGCA
GCAGGTATCACTCACGGTATGGACGAACTTTATAAATAAGGATCCTACTCGAGCCACATAGATGGCGTCGCTAGTATTAAATG
CATATTATTTTTATATAGTACCAACCTTCAAATGATTCCCTATAGCTTGTAAATTCTATCATAATTGTGGTTTCAAAATCGGC
TCCGTCGATACTATGTTATACGCCAACTTTGAAAACAACTTTGAAAAAGCTGTTTTCTGGTATTTAAGGTTTTAGAATGCAAG
GAACAGTGAATTGGAGTTCGTCTTGTTATAATTAGCTTCTTGGGGTATCTTTAAATACTGTAGAAAAGAGGAAGGAAATAAT
AAATGGCTAAAATGAGAATATCACCGGAATTGAAAAAACTGATCGAAAAATACCGCTGCGTAAAAGATACGGAAGGAATGTC

TCCTGCTAAGGTATATAAGCTGGTGGGAGAAAATGAAAACCTATATTTAAAAATGACGGACAGCCGGTATAAAGGGACCACCT
ATGATGTAGAACGGGAAAAGGACATGATGCTATGGCTGGAAGGAAAGCTGCCTGTTCCAAAGGTCCTGCACTTTGAACGGCAT
GATGGCTGGAGCAATCTGCTCATGAGTGAGGCCGATGGCGTCCTTTGCTCGGAAGAGTATGAAGATGAACAAAGCCCTGAAAA
GATTATCGAGCTGTATGCGGAGTGCATCAGGCTCTTTCACTCCATCGACATATCGGATTGTCCCTATACGAATAGCTTAGACA
GCCGCTTAGCCGAATTGGATTACTTACTGAATAACGATCTGGCCGATGTGGATTGCGAAAACTGGGAAGAAGACACTCCATTT
AAAGATCCGCGCGAGCTGTATGATTTTTTAAAGACGGAAAAGCCCGAAGAGGAACTTGTCTTTTCCCACGGCGACCTGGGAGA
CAGCAACATCTTTGTGAAAGATGGCAAAGTAAGTGGCTTTATTGATCTTGGGAGAAGCGGCAGGGCGGACAAGTGGTATGACA
TTGCCTTCTGCGTCCGGTCGATCAGGGAGGATATCGGGGAAGAACAGTATGTCGAGCTATTTTTTGACTTACTGGGGATCAAG
CCTGATTGGGAGAAAATAAAATATTATATTTTACTGGATGAATTGTTTTAGTACCTAGATTTAGATGTCTAAAGAATTGATG
CTTTAACTACATGCTTTTTAGACATCTAATCTTTTCTGAAGTACATCCGCAACTGTCCATACTCTGATGTTTTATATCTTTTCT
AAAAGTTCGCTAGATAGGGGTCCCGAGCGCCTACGAGGAATTTGTATCGGGGTATAGATCTAAAACGAATTTTAACAAAAAAA
TGTGTTTTTCTTTGTTCTCGAGCCCGGGGCTAGCACTAGTAGACCGGGGACTTATCAGCCAACCTGTTATTTGGAGCCCCGAGG
AAATCAAGTCAGCGAAAATCTCCAGCAGGCGGCCAGAGAAGCGTCGCCCTACTTAACTTACCTTGGCGCATTCAGCTTCCAGGC
ACAGCGGAACGGAACCTTAGTCGCACCGCCTTTAACGAATTTAAGGAGCATTACAGAAAGTCAAAATACAACATTGATGATGA
TTATAACGAACCTAGAAAACCAGGCATTCAGCGATGAACTTGGCCGGATCCTTTTGAACGACGAAACTGTAAAAAGACGGCTT
CTAAATGAAATAGTCGAGAATGCCAGAAGATATGGCTTCCGTGACATTCATTTCGACTTTGAATATTTGCGGCCCCAGGATAG
AGAGGCCTATAATCAATTCCTCCGCGAAGCAAGGGATCTTTTCCATCGAGAGGGCTTAGAAATTTCTACGGCTCTTGCTCCTA
AAACAAGTGCAACACAGCAGGGCAGGTGGTATGAAGCTCATGATTACAGGGCACATGGCGAAATTGT

*yfmG::*TnJM1

CGGGAGGTTATCGTAATTTGACTCTATTAATGCAAGATCATAAATCAATTTACAATAAAGATATAGAAATATTAATTGAAGT
TGAAGAACTTATTACTTCTGTTCCAGAAAATAAAAAAAATGATTTTAAGAGTTTAATCGATAATAGAATTATGACTCTACTA
AATAAAATTCAACACCCTGTAGCAGCGGAAAATGTGTTTGGGATGTGTATTGCTTATCCGAATACTTACATAAGGTATAGATT
AATTGATTTCGTTGAAAAATGGGTACCTTATTTCTCTGCTGTAGAAACTATAATCAATCTAACCCAGGATCCGGATGATTTAG
TTTCTTTTAAAGCTATGGATGTCTGTGCAAATCATAAAATTGAAGAATCTGTAGCATATTTGTCTTCGATTATTGATGATGTC
CGGGAGAGTATAACAGGTTGGCTGATAAGTCCCCGGTCTGGTCGACTAAGTGAGGATTAACTAATAAGGAGGACAAACATGTC
AAAAGGAGAAGAACTTTTTACAGGTGTAGTACCTATCTTGGTTGAATTGGATGGTGATGTTAACGGTCACAAATTTTCTGTAC
GTGGTGAAGGTGAAGGTGATGCAACTAACGGTAAATTGACACTTAAATTCATTTGTACAACTGGAAAACTTCCTGTTCCTTGG
CCTACTCTTGTTACAACATTGACATATGGAGTACAATGTTTTTCACGTTATCCTGATCATATGAAACGTCACGATTTTTTTAA
ATCTGCTATGCCAGAAGGTTATGTACAAGAACGTACAATTTCATTTAAAGATGACGGAACATATAAAACACGTGCTGAAGTAA
AATTCGAAGGTGACACTCTTGTTAATCGTATCGAATTGAAAGGAATCGATTTCAAAGAAGATGGTAACATTTTGGGACACAAA
CTTGAATACAACTTCAACTCTCATAATGTTTATATCACAGCTGACAAACAAAAAAACGGTATTAAAGCTAATTTTAAAATTCG
TCACAATGTTGAAGATGGATCTGTTCAATTGGCTGATCATTATCAACAAAATACACCAATCGGAGACGGACCAGTATTGCTTC
CAGATAACCACTACCTTTCTACTCAATCAGTTCTTTCAAAAGATCCTAACGAAAAACGTGACCATATGGTACTTCTTGAATTT
GTTACAGCAGCAGGTATCACTCACGGTATGGACGAACTTTATAAATAAGGATCCTACTCGAGCCACATAGATGGCGTCGCTAG
TATTAAATGCATATTATTTTTATATAGTACCAACCTTCAAATGATTCCCTATAGCTTGTAAATTCTATCATAATTGTGGTTTC
AAAATCGGCTCCGTCGATACTATGTTATACGCCAACTTTGAAAACAACTTTGAAAAAGCTGTTTTCTGGTATTTAAGGTTTTA
GAATGCAAGGAACAGTGAATTGGAGTTCGTCTTGTTATAATTAGCTTCTTGGGGTATCTTTAAATACTGTAGAAAAGAGGAA
GGAAATAATAAATGGCTAAAATGAGAATATCACCGGAATTGAAAAAACTGATCGAAAAATACCGCTGCGTAAAAGATACGGA
AGGAATGTCTCCTGCTAAGGTATATAAGCTGGTGGGAGAAAATGAAAACCTATATTTAAAAATGACGGACAGCCGGTATAAA
GGGACCACCTATGATGTAGAACGGGAAAAGGACATGATGCTATGGCTGGAAGGAAAGCTGCCTGTTCCAAAGGTCCTGCACTT
TGAACGGCATGATGGCTGGAGCAATCTGCTCATGAGTGAGGCCGATGGCGTCCTTTGCTCGGAAGAGTATGAAGATGAACAAA
GCCCTGAAAAGATTATCGAGCTGTATGCGGAGTGCATCAGGCTCTTTCACTCCATCGACATATCGGATTGTCCCTATACGAAT
AGCTTAGACAGCCGCTTAGCCGAATTGGATTACTTACTGAATAACGATCTGGCCGATGTGGATTGCGAAAACTGGGAAGAAGA
CACTCCATTTAAAGATCCGCGCGAGCTGTATGATTTTTTAAAGACGGAAAAGCCCGAAGAGGAACTTGTCTTTTCCCACGGCG
ACCTGGGAGACAGCAACATCTTTGTGAAAGATGGCAAAGTAAGTGGCTTTATTGATCTTGGGAGAAGCGGCAGGGCGGACAAG
TGGTATGACATTGCCTTCTGCGTCCGGTCGATCAGGGAGGATATCGGGGAAGAACAGTATGTCGAGCTATTTTTTGACTTACT
GGGGATCAAGCCTGATTGGGAGAAAATAAAATATTATATTTTACTGGATGAATTGTTTTAGTACCTAGATTTAGATGTCTAA
AGAATTGATGCTTTAACTACATGCTTTTTAGACATCTAATCTTTTCTGAAGTACATCCGCAACTGTCCATACTCTGATGTTTT
ATATCTTTTCTAAAAGTTCGCTAGATAGGGGTCCCGAGCGCCTACGAGGAATTTGTATCGGGGTATAGATCTAAAACGAATTT
TAACAAAAAAATGTGTTTTTCTTTGTTCTCGAGCCCGGGGCTAGCACTAGTAGACCGGGGACTTATCAGCCAACCTGTAGCTA
CCCTAAAAAACCTGTGGGATTAGGGGCCCAAAAAGTATTAAGTACATTATTAGATATATTCGGTGTTGAAAAACATGAAGAGC
TAGTAATGCTAAAAAATTATTTTGATCAAAATGGTATACTTCCTAATAACTTTGACTTTGAAGAAAAAATTCCTCAAAGTCTA
ATAGAGGAATTCGAAAAAACAGAAGAAGATGGGATGATTTTAATTCCGGGTGGCTTTTTTGAATTCGGTTTAAATGAAAATG
AAATTCCTGACAAAACATTTAATTGGAAAGATGCTGTTCCTAGACAAAAAGTATGGTTACCTCCATTTTTTATCGATAAGTAT
CCTGTTACAAATAAAGATTATGATATCTTTACCGAGTTCATAGAGGAAAATGGGCATATATTCTGTCATCCTAATGAACCGCA
GAATAAACAACATAGAAGAAATACATATTGGGATGATAGGTATTTAGATAATCATCCCGTTACAGGTATAGATTTTTATGA

Supplementary Information S2. Representative movies of the phase contrast and epifluorescence (reporting *sfgfp(Sp*) expressed from the TnJM1 transposon inserted into the indicated genes) channels of reconstructed mutants grown on MOPS agarose pads. Due to differences in fluorescence expression among the isolated clones, image brightness was adjusted for each mutant separately and fluorescence intensity can therefore not be compared between videos. https://data.mendeley.com/datasets/52bhwgf3wh/1 (DOI: 10.17632/52bhwgf3wh.1)

References

[1] W. Overkamp *et al.*, “Benchmarking various green fluorescent protein variants in Bacillus subtilis, Streptococcus pneumoniae, and Lactococcus lactis for live cell imaging,” *Appl. Environ. Microbiol.*, vol. 79, no. 20, pp. 6481–6490, 2013, doi: 10.1128/AEM.02033-13.

[2] E. R. Pozsgai, K. M. Blair, and D. B. Kearns, “Modified mariner transposons for random inducible-expression insertions and transcriptional reporter fusion insertions in Bacillus subtilis,” *Appl. Environ. Microbiol.*, vol. 78, no. 3, pp. 778–785, 2012, doi: 10.1128/AEM.07098-11.
